# Identification of microRNAs controlling hepatic mRNA levels for metabolic genes during the metabolic transition from embryonic to posthatch development in the chicken

**DOI:** 10.1186/s12864-017-4096-5

**Published:** 2017-09-05

**Authors:** Julie A. Hicks, Tom E. Porter, Hsiao-Ching Liu

**Affiliations:** 1 Department of Animal Science, North Carolina State University, Polk Hall 232D, Box 7621, Raleigh, NC 27695 USA; 2 Department of Animal and Avian Sciences, University of Maryland, College Park, MD 20742 USA

**Keywords:** Chicken, Genomics, miRNA, Metabolism, Liver

## Abstract

**Background:**

The transition from embryonic to posthatch development in the chicken represents a massive metabolic switch from primarily lipolytic to primarily lipogenic metabolism. This metabolic switch is essential for the chick to successfully transition from the metabolism of stored egg yolk to the utilization of carbohydrate-based feed. However, regulation of this metabolic switch is not well understood. We hypothesized that microRNAs (miRNAs) play an important role in the metabolic switch that is essential to efficient growth of chickens. We used high-throughput RNA sequencing to characterize expression profiles of mRNA and miRNA in liver during late embryonic and early posthatch development of the chicken. This extensive data set was used to define the contributions of microRNAs to the metabolic switch during development that is critical to growth and nutrient utilization in chickens.

**Results:**

We found that expression of over 800 mRNAs and 30 miRNAs was altered in the embryonic liver between embryonic day 18 and posthatch day 3, and many of these differentially expressed mRNAs and miRNAs are associated with metabolic processes. We confirmed the regulation of some of these mRNAs by miRNAs expressed in a reciprocal pattern using luciferase reporter assays. Finally, through the use of yeast one-hybrid screens, we identified several proteins that likely regulate expression of one of these important miRNAs.

**Conclusions:**

Integration of the upstream regulatory mechanisms governing miRNA expression along with monitoring the downstream effects of this expression will ultimately allow for the construction of complete miRNA regulatory networks associated with the hepatic metabolic switch in chickens. Our findings support a key role for miRNAs in controlling the metabolic switch that occurs between embryonic and posthatch development in the chicken.

**Electronic supplementary material:**

The online version of this article (10.1186/s12864-017-4096-5) contains supplementary material, which is available to authorized users.

## Background

The major energy source for chicken embryos is the yolk, which consists of lipoproteins used for fatty acid oxidation by the liver [1]. The majority of this lipid metabolism happens during the last week of embryonic development, during which ~80% of yolk lipids are absorbed [1]. Residual yolk lipids are rapidly depleted within 3 days of hatch [1]. The hatchlings’ metabolism must then quickly switch to a carbohydrate-based energy source (corn-based feed). Proper and prompt metabolic switching is essential for efficient growth of the chicken. This is evidenced by the fact that delayed feeding of newly hatched chicks for 48 h retards lipogenic gene expression and reduces long-term body weight gain [2].

Most of the energy required for embryo development is derived from the metabolism of yolk lipids [3–5], and gluconeogenesis occurs primarily from metabolism of glycerol, a product of lipid metabolism [6]. Chicken embryos grow exponentially during the final week of incubation, with about 90% of the total energy for growth derived from *β*-oxidation of yolk fatty acids [4]. In contrast, energy for posthatch growth is derived primarily from corn-based diets, although residual yolk remains a source of energy for the first 48–72 h [1, 7, 8]. Transfer of lipids from the yolk to the embryo increases dramatically from embryonic day (**E**) 15 until hatching [1]. It has been estimated that 1 g of lipids/day is transferred to the embryo between E20 and E21 [1]. In birds, the liver, not adipose, is the major site of embryonic lipid utilization and posthatch lipid synthesis [9]. Thus, the most dynamic changes in lipid composition of embryonic tissues occur in the liver [1]. By E19, hepatic lipid concentrations account for 5% of total lipids absorbed from the yolk [1]. A few days prior to hatching, the yolk begins to be absorbed into the embryo and remains a nutrient source for the newly hatched chick until the initiation of feed consumption [2]. The transition from yolk lipid metabolism to metabolism of carbohydrates in feed is essential for efficient growth of the chicken, and this metabolic switch is necessary to thrive on corn-based diets.

A few of the processes underlying the metabolic switch at hatching in chickens have been characterized. For example, the final week of embryonic development is associated with a dramatic accumulation of lipid and cholesterol in the liver due to increased yolk lipid uptake [3, 10], and hepatic lipogenic capacity increases dramatically after hatching [1]. Changes in hepatic gene expression pre- and post-hatching have been characterized using DNA microarrays, and results indicate that mRNA levels for genes involved in lipid metabolism are high during embryonic development and decrease dramatically after hatching, while expression of genes involved in lipogenesis is low prior to hatching and increases substantially after hatching [11–13]. Some of the genes with higher embryonic expression include genes involved in lipid metabolism (acetyl-CoA acetyltransferase 2), gluconeogenesis (phosphoenolpyruvate carboxykinase 2), and glycolysis (aldolase, fructose-bisphosphate A), while those expressed at higher levels after hatching include genes involved in lipogenesis (fatty acid synthase, ***FASN***; malic enzyme, ***ME***) and cholesterol synthesis (HMG CoA synthase, ***HMGCS***). Other differentially expressed pre- and post-hatching genes encode for transcription factors known to regulate lipid metabolism and lipogenesis, including peroxisome proliferator-activated receptor-α (***PPARA***) and *PPARG*. Hepatic levels of *PPARA* mRNA, which regulates expression of genes involved in *β*-oxidation of fatty acids, are higher in embryos. In contrast, levels of *PPARG* mRNA, which regulates expression of genes involved in lipogenesis, increase after hatching. Thus, the metabolic switch at hatching involves decreased lipid metabolism and increased lipogenesis by the liver, and this switch is likely regulated, at least in part, at the level of transcription.

At least some gene expression changes after hatching are regulated by feed intake, as their up- or down-regulation is delayed in chicks with postponed access to feed for 48 h after hatch [2]. These included *ME*, *FASN*, ATP citrate lyase, stearoyl-Coenzyme A desaturase (***SCD***), acetyl-CoA carboxylase alpha (***ACACA***), 3-hydroxy-3-methylglutaryl-Coenzyme A reductase, lipoprotein lipase, fatty acid desaturase 1 (***FADS1***), ***FADS2***, ELOVL fatty acid elongase 6 (***ELOVL6***), *ELOVL5*, *ELOVL1*, carnitine palmitoyltransferase 1A, sterol regulatory element-binding protein-1 (***SREBP1***), *SREBP-2*, *PPARA*, *PPARG*, and PPARG coactivator 1 alpha. In each case, typical gene expression changes were delayed by 48 h, after initial feed intake in this delayed feeding paradigm. Thus, posthatch changes in gene expression related to fatty acid oxidation, lipogenesis, and cholesterol synthesis are likely regulated by the intake of high carbohydrate feed, its metabolites, or ensuing hormonal changes (e.g., increased insulin, decreased glucagon), rather than hatching or yolk depletion.

Mature microRNAs (**miRNA**) are single-stranded, small, noncoding RNAs that regulate gene expression post-transcriptionally by either preventing translation and/or promoting target mRNA degradation in a process referred to as RNA interference (**RNAi**) [14]. MiRNAs have been shown to participate in many metabolic processes, including regulation of lipid trafficking and levels of circulating cholesterol in mammals [15]. We have previously shown that many miRNAs are dynamically expressed in the embryonic chicken liver and that these miRNAs can regulate genes associated with lipid metabolism [16]. However, little is known about hepatic miRNA-mediated regulation during the metabolic switch of newly hatched chickens. We hypothesized that miRNAs play important roles in the metabolic switch that is essential to efficient growth of chickens. We have previously identified known and novel miRNAs expressed in chicken liver on E15 and E20 [16]. Potential targets of the novel miRNAs were predicted and confirmed using a retroviral system. One novel miRNA identified, *nc*-*miR-33* was shown to regulate expression of *FASN*. Thus, we have already identified one miRNA likely involved in regulating lipogenesis in the chicken liver. This report was the first to discover a miRNA involved in regulation of metabolic pathways by the chicken liver. Subsequently, chicken *miR-33* was shown to repress expression of the chicken fat mass and obesity associated gene [17]. Recently, inverse relationships were identified between several miRNAs and their predicted mRNA targets in metabolic pathways in growth hormone (**GH**)-treated chicken hepatocytes [18]. This finding indicates that certain miRNAs and their predicted targets are under hormonal control in the chicken liver. However, functional regulation of the predicted mRNA targets by the miRNAs identified was not confirmed.

A comprehensive analysis of miRNA involvement in metabolic regulation in the chicken liver has not been reported. The present study was designed to characterize expression profiles for mRNA and miRNA in the liver during the metabolic switch from embryonic to posthatch development. Reciprocal changes in miRNA levels and levels of their predicted target mRNA were identified. Predicted target mRNAs for genes involved in metabolic pathways were confirmed using retroviral expression of miRNAs and luciferease reporter assays for predicted target mRNAs. Potential transcription factor proteins controlling expression of one of the confirmed regulatory miRNAs were identified using yeast-one hybrid assays.

## Methods

### Differential expression of miRNA and mRNA

Hepatic miRNA and mRNA expression profiles were developed from four birds at each age of development, embryonic day (**E**) 18, E20, posthatch day (**D**) 0, D1, and D3, in specific pathogen free (SPF) Leghorn chickens (layers). SPF chickens were chosen for this study to minimize environmental impacts on gene expression to ensure identification of gene expression changes only associated with metabolic changes. D0 samples were collected before initiation of feeding. D1 and D3 birds received ad libitum access to commercial starter ration and water. Fertilized eggs were obtained from Charles River Laboratories (Wilmington, MA) and incubated in a humidified incubator (37.5 C, 60% relative humidity, rotation every hour). Liver samples were collected and snap frozen and stored at −80 °C. All animal procedures were approved by the Institutional Animal Care and Use Committee at North Carolina State University. Total RNA was isolated using Tri-Reagent (Sigma). RNA was purified following the manufacturer’s instructions with the exception that RNA was precipitated overnight at −20 °C. RNA was quantified using a nanodrop ND-1000 spectrophotometer, and quality was assessed using agarose gel electrophoresis. For miRNA-seq, small RNAs were enriched from total RNA using a miRVana miRNA isolation kit (Ambion), and samples were subjected to on-column DNase treatment. Quality of all RNA samples was then assessed using an Agilent Technologies 2100 Bioanalyzer with a high sensitivity RNA chip. All RNA samples had RIN values >9.

Small RNA libraries for each developmental time point were generated from pooled (4 birds per time point) small RNA samples using a TruSeq Small RNA sample preparation kit (Illumina) and barcode indices following the manufacturer’s instructions. For each library, 1 μg of enriched small RNAs was used. The quality and quantity of the libraries were assessed on an Agilent Technologies 2100 Bioanalyzer using a high sensitivity DNA chip. Each library was diluted to 10 nM using 10 mM Tris-HCl (pH 8.5) and then 4 μl of each library were pooled. Pooled DNA was sequenced on a single lane of an Illumina Genome Analyzer IIx (GAIIx) (NCSU Genomic Sciences Laboratory).

RNA-seq (mRNA) libraries were generated for individual birds (*n* = 4) at each developmental time point using a TruSeq RNA library preparation kit v2 (Illumina) and barcode indices following the manufacturer’s instructions. The quality and quantity of the libraries were assessed on an Agilent Technologies 2100 Bioanalyzer using a high sensitivity DNA chip. Equal molar amounts of the libraries were pooled (100 ng total per pool) and 50 bp single-end sequenced at DHMRI (Kannapolis, NC) using an Illumina HiSeq 2500.

All FASTQ sequencing files have been deposited to the NIH Short Read Archive (accession numbers SAMN06651251-SAMN06651275). All sequencing data processing and analyses were performed using CLC genomics workbench (Qiagen). Briefly, FASTQ files were imported into the CLC genomics workbench software. The NGS trim tool was used to remove any residual adaptor sequences and/or low quality sequences (Phred < 20). Reads were then mapped to the *Gallus gallus* reference genome (Gallus_gallus-5.0) and normalized using the transformation and normalization tool. Expression analysis of the small RNA libraries was carried out using the small RNA analysis suite, and mRNA libraries were analyzed using the RNA-seq analysis suite. Specifically, differential expression was determined using the “Empirical analysis of DGE” tool, which implements the “exact test” developed by Robinson and Smyth [19] and a FDR corrected *p*-value cutoff of 0.05. Pairwise comparisons were made between developmental time points. Ingenuity Pathway Analysis (Qiagen) was then performed on the differential expression data using the integrated plugin.

### Application of an RNAi system to verify miRNA target sequence

Potential target genes for selected significantly differentially expressed miRNA were identified using the *Gallus gallus* Unigene database (NCBI) and the miRanda algorithm (version 3.3; http://www.microrna.org) with the following parameter settings: score threshold >130 and free energy threshold < −16 kCal/mol. The list of potential target genes was further filtered using the following higher stringency methods: (1) a match between nucleotides 2–8 of the miRNA with the target sequence or (2) a match between nucleotides 2–7 and 13–16 of the miRNA with the target sequence (G:U wobble tolerance) and (3) miRNA binding sites must lie within the 3’UTR. For each potential target gene, the 3’UTR flanking the miRNA binding site(s) were PCR amplified from chicken genomic DNA using gene-specific primers. Each PCR product was cloned into the 3′ UTR of the *Renilla* luciferase reporter gene in the psiCHECK-2 vector (Promega) using the NotI and XhoI restriction sites. The psiCHECK-2 vector contains both the Renilla luciferase reporter gene to monitor small RNA targeting as well as the independent firefly luciferase reporter gene to account for any differences in transfection efficiency.

#### Construction of RCAS expressing chicken miRNA vectors

The RCASBP(A)-miR vector previously described by Chen et al. [20] was utilized for ectopic miRNA expression or for expression of a scrambled control sequence (SC). This system utilizes gateway cloning technology to insert and express a miRNA hairpin from the retroviral LTR region. The scrambled control sequence is expressed in the context of the *miR-30a* hairpin. MiRNA hairpin primers were designed based on the chicken precursor sequences for each miRNA. PAGE-purified forward and reverse primers (Invitrogen) were mixed at a final concentration of 1 μM, denatured at 95 °C for 20 s and annealed at RT. The DNA fragment was then cloned into the pENTR3C-miR-SphNgo vector at the SphI and NgoMIV restriction sites. To generate the RCASBP(A)-*miR* vector, a recombination between the pENTR3C-*miR* entry vector and RCASBP(A)-YDV gateway destination vector was performed using a LR clonase kit (Invitrogen).

#### Dual luciferase reporter assay

DF1 cells were infected with either RCAS-*gga-let-7c*, RCAS-*gga-miR-20b*, RCAS-*gga-miR-183* or RCAS-*SC* (M.O.I. of 1) and maintained in a 96-well plate in RPMI 1640 with 1% heat-inactivated FBS, L-glutamine, penicillin (100 U/ml), streptomycin (100 μg/ml), and fungizone (4 μg/ml), at 37 °C with 5% CO_2_. At 3 dpi, each psiCHECK-2 target construct (100 ng) was transfected (in triplicate) into the DF1 cells using FuGENE 6 (Promega). Forty-eight hours post-transfection, cells were lysed in Passive Lysis Buffer (Promega), and firefly and *Renilla* luciferase activities were then assessed using the Dual-Luciferase Reporter Assay System (Promega) and a VictorLight 1420 luminescence counter (PerkinElmer). Normalized luciferase activity was calculated from the *Renilla/*firefly signal ratio. Analysis of variance (*p* < 0.05) was used to determine repression of the *Renilla* reporter gene by a given miRNA by comparing the relative luciferase activity between cells infected with an RCAS expressing the miRNA and the RCAS expressing the scrambled control sequence. The assay was independently repeated to confirm the results.

### Yeast one-hybrid system examination of regulation of chicken miRNA expression

Yeast one-hybrid analyses were carried out using the Matchmaker Gold yeast one-hybrid system (Clontech) as directed by the manufacturer. The upstream region (~4 kb) of *gga-miR-20b* was obtained from Ensembl (http://useast.ensembl.org/index.html), and promoter-like elements upstream of *gga-miR-20b* were predicted using PROSCAN [21]. The region containing all potential regulatory elements (~350 bp) was cloned into the pAbAi vector using KpnI and XhoI and sequenced. A bait strain containing the *gga-miR-20b* promoter cassette was generated following the manufacturer’s instructions. Briefly, Y1H Gold yeast were transformed with 1 μg of linearized (using BstBI) pAbAi-*gga-miR-20b*-pro vector and yeast with positive cassette integration were selected using SD-Ura media. Y1H Gold-pAbAi-*gga-miR-20b*-pro yeast were then tested on SD-Ura containing a range of Aureobasidin A (Aba) concentrations to determine the optimal Aba concentration for library screening (350 ng/mL). The cDNA library was produced from liver tissues from three birds each at E18, E20, D0, D1, and D3. For cDNA production, mRNA was purified using a NucleoTrap mRNA kit (Clontech). One microgram of mRNA from each sample was pooled, and one microgram of pooled mRNA was used for reverse-transcription using SMART RT (Clontech). SMART cDNA was then used in long-distance PCR to produce a double-stranded cDNA library following the manufacturer’s instructions. Library quality was assessed using gel electrophoresis. The library was purified using a CHROMA SPIN + TE-400 column (Clontech) and concentrated (ethanol/sodium acetate precipitation) as directed by the manufacturer. The cDNA library (4.9 μg) was transformed into the Y1H Gold-pAbAi-*gga-miR-20b*-pro yeast following the manufacturer’s instructions and screened on SD-Leu media containing 350 ng/mL Aba. Positive colonies were further selected by re-plating (3X) on SD-Leu-350 ng/mL Aba. Approximately 6.2 million colonies were screened, and 48 positive clones (i.e. Aureobasidin A toxin resistant) were sequenced. Sequences were then mapped to the *Gallus gallus* refseq database (NCBI) to determine their identity.

## Results

### miRNA- and RNA-seq library characteristics

For the small RNA libraries, trimmed mappable reads ranged from 1,265,193 to 1,580,894, and for the mRNA libraries trimmed mappable reads ranges from 5,454,096 to 12,824,291. The number of unique miRNAs detected (CPM ≥ 30) ranged from 96 to 109, and the number of unique annotated transcripts (RPKM ≥ 30) ranged from 1186 to 1722. Tables of all identified miRNAs (Additional file 1) and transcripts (Additional file 2) are provided as additional files. The expression patterns of a select group of miRNAs and mRNAs were confirmed using RT-qPCR (Additional file 3). The miRNA and mRNA selected for confirmation by RT-qPCR were based on their role or predicted role in metabolic pathways. In all cases RNAseq results were confirmed by RT-qPCR.

### Changes in hepatic miRNA expression during chicken liver development

Stepwise comparisons of miRNA expression profiles of hepatically expressed miRNAs in late stage embryonic and early post-hatch chicks revealed that the most dramatic changes in mRNA expression occurred in the first days post-hatch. Between E18 and E20 14 miRNAs had a greater than two-fold change in expression. Between E20 and D0 the number of differentially expressed miRNA dropped to five. The D0-D1 comparison identified 19 differentially miRNAs, while the D1-D3 had the biggest change with 23 differentially expressed miRNAs. Integration of in silico miRNA prediction, miRNA target databases (targetscan), and the IPA microRNA target filter, revealed that many of these differentially expressed miRNAs likely target mRNA with known functions in metabolic pathways, which were also found to be differentially expressed in this study (Additional file 4).

### Expression dynamics of genes during chicken liver development

Liver transcriptome changes (>2-fold, *p* < 0.05) during the late embryonic/early post-hatch stages of chicken development identified here included 340 genes at the E18-E20 transition, 227 genes at the E20-D0 transition, 153 genes at the D0-D1 transition and 184 genes at the D1-D3 transition. IPA analysis of these differentially expressed genes revealed that they are involved in many different developmental and metabolic processes (Table 1). Comparison of the E18 (pre-switch) and D3 (post-switch) time-points revealed that levels of 823 mRNA were altered (>2-fold, p < 0.05) in the liver. The top five regulator effect networks included: (1) conversion of lipid, metabolism of sterol, (2) conversion of lipid, metabolism of cholesterol, (3) conversion of fatty acid, (4) adipogenesis of cells, conversion of lipid, and (5) metabolism of cholesterol, synthesis of sterol. The top molecular and cellular function was lipid metabolism.

**Table 1:** Top cellular pathways and functions associated with liver development in chickens

Time point Comparison	Top Canonical Pathways	Top Upstream Regulators	Molecular and Cellular Functions	Physiological System Development and Function
E18-E20	protein ubiquitination pathway; unfolded protein response; purine nucleotides de Novo Biosynthesis II; FXR/RXR Activation; Aryl hydrocarbon Receptor signaling	TP53; beta-estradiol; XBP1; PPARA; HNF4A	cell death and survival; cellular growth and proliferation; amino acid metabolism; small molecule biochemistry; lipid metabolism	organismal survival; digestive system development and function; organ morphology; connective tissue development and function
E20-D0	FXR/RXR activation; LXR/RXR activation; LPS/IL-1 mediated inhibition of RXR function; acute phase response signaling; coagulation system	TP53; PPARA; beta-estradiol; methylprednisolone; MYC	lipid metabolism; small molecule Biochemistry; molecular transport; cell death and survival; amino acid metabolism	organismal survival; digestive system development and function; hepatic system development and function; organ morphology; organismal development
D0-D1	EIF2 signaling; LPS/IL-1 mediated inhibition of RXR function; mitochondrial dysfunction; acute phase response signaling; FXR/RXR activation	PPARA; HNF4A; methylprednisolone; pirinixic acid; MYC	amino acid metabolism; small molecule biochemistry; lipid metabolism; molecular transport; carbohydrate metabolism	digestive system development and function; hepatic system development and function; organ morphology; organismal development; tissue morphology
D1-D3	Super pathway of cholesterol biosynthesis; cholesterol biosynthesis I; cholesterol biosynthesis II (via 24, 25-dihydrolanosterol); cholesterol biosynthesis III (via Desmosterol); mitochondrial dysfunction	PPARA; SREBF1; SCAP; POR; SREBF2	lipid metabolism; molecular transport; small molecule biochemistry; vitamin and mineral metabolism; energy production	digestive system development and function; hepatic system development and function; organ morphology; organismal development; connective tissue development and function

### MiRNA and metabolic gene hepatic expression differences between the pre- and post-hatch chickens

**Fig. 1:**
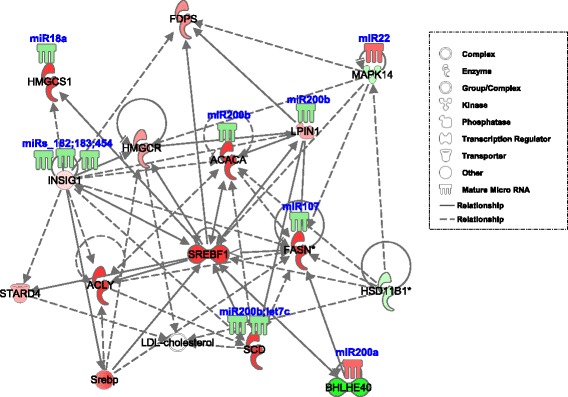
Differential hepatic expression of a SREBF1 miRNA regulatory network between E18 and D3 chickens. SREBF1 is a transcription factor recognizing sterol regulatory element-1 sites and regulates fatty acid and cholesterol synthesis. Red is increased expression in the D3 liver compared to E18, and Green is decreased expression. Many SREBF1 regulated genes are also regulated by miRNAs, including *let-7c*, *miR-200b*, *miR-107*, and *miR-18a*

**Fig. 2:**
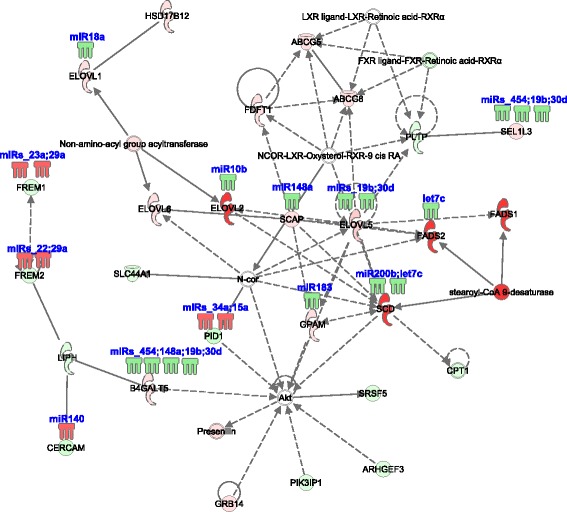
Differential hepatic expression of a lipid metabolism network involving *FADS2* and *SCD* and associated miRNA between E18 and D3 chickens. Red is increased expression in the D3 liver compared to E18, and Green is decreased expression. *FADS2*, *SCD* and other genes in this network are predicted targets of miRNA, including *let-7c* and *miR-183*

**Fig. 3:**
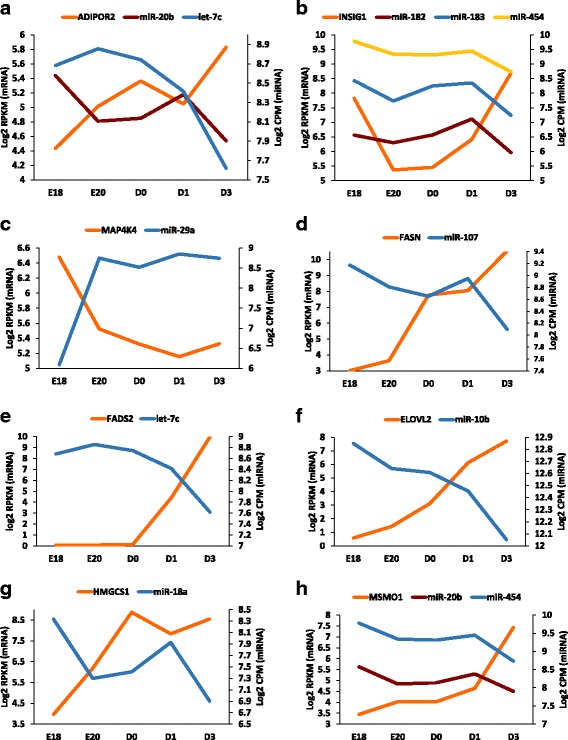
Examples of reciprocal expression of metabolic genes and some their targeting miRNAs during the metabolic switch in developing chickens. Levels of mRNA and miRNA in liver samples from E18, E20, D0, D1 and D3 chickens were determined by RNAseq and small RNAseq, respectively. Levels of mRNA are expressed as the log2 of the RPKM. Levels of miRNA are presented as the log2 of the CPM. **a** ADIPOR2 is targeted by *miR-20b* and *let-7c*, **b** INSIG1 is targeted by *miR-182*, *miR-183*, and *miR-454*, **c** MAPK4K4 is targeted by *miR-29a*, **d** FASN is targeted by *miR-107*, **e** FADS2 is targeted by *let-7c*, **f** ELOVL2 is targeted by *miR-10b*, **g** HMGCS1 is targeted by *miR-18a*, and **h** MSMO1 is targeted by *miR-20b* and *miR-454*

Comparison of the E18 (pre-metabolic switch) and D3 (post-metabolic switch) time points had the largest differences in expression of miRNAs and metabolism-associated genes. This comparison revealed that 31 miRNAs were differentially expressed (>2-fold) in the liver, while levels of 823 mRNA were altered (>2-fold, *p* < 0.05). Integration of the miRNA and mRNA expression data in conjunction with the Ingenuity Pathway Analysis (**IPA**) knowledgebase revealed that several of the differentially expressed miRNAs likely regulate pathways associated with the metabolic switch. MiRNA target prediction was based on sequence identity between the mature miRNA and the 3′-UTR of the mRNA. Figure 1 depicts a regulatory network of genes controlling lipid metabolism, including *INSIG1* and *SREBF1*. SREBF1 is a transcription factor recognizing sterol regulatory element-1 sites and regulates fatty acid and cholesterol synthesis. Many SREBF1-regulated genes are also regulated by miRNAs, including *let-7c*, *miR-200b*, *miR-107*, and *miR-18a*. Figure 2 illustrates a lipid metabolism network involving *FADS2* and *SCD* and associated miRNA. *FADS2*, *SCD* and other genes in this network are predicted targets of miRNA, including *let-7c* and *miR-183.* Additional gene networks with predicted regulatory miRNAs are provided in Additional file 4. Expression profiles of selected mRNA in metabolic pathways determined from the IPA analysis that were predicted to be regulated by miRNA and of the miRNAs predicted to regulate these genes are presented in Fig. 3.

### Target prediction analysis and validation of differentially expressed miRNAs

**Fig. 4:**
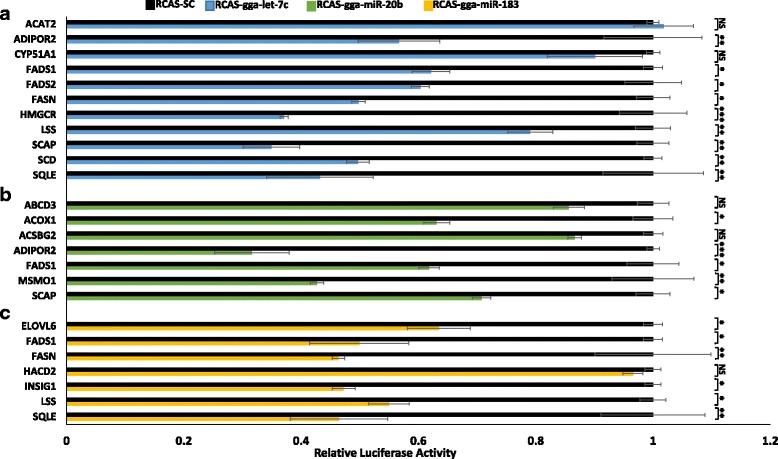
Validated chicken target genes for *let-7c*, *miR-20b*, and *miR-183*. Luciferase assays for (a) let-7c, (b) miR-20b, (c) miR-183, and a scrambled sequence (SC) for target gene validation are shown. *: p < 0.05, **: *p* < 0.01, ***: *p* < 0.001, NS: not significant

To further explore miRNA regulatory networks associated with the metabolic switch, we identified potential metabolic mRNA targets for *let-7c*, *miR-20b*, and *miR-183* using in silico target prediction in combination with IPA pathway analysis. Unfortunately, it is not feasible to validate targets for all differentially expressed miRNAs. These particular miRNA were selected based on their hepatic expression patterns and their propensity for targeting multiple metabolic pathway-associated genes differentially expressed in this study (Table 2). This analysis revealed that all three miRNAs, (*let-7c*, *miR-20b*, and *miR-183*), potentially regulate a number of genes associated with lipid metabolism and carbohydrate metabolism (Table 2). However, these in silico predicted targets have not yet been confirmed. To that end, we utilized a retroviral-based miRNA expression system to experimentally validate a select group of predicted target mRNAs. Genes were selected for validation based on the following criteria: (1) the thermodynamics of predicted miRNA-target site interactions; (2) known metabolic functions of the predicted targets; (3) significant up-regulated hepatic expression between E18 and D3 (inverse relationship with the selected miRNAs); and (4) number of miRNA binding sites. For *let-7c*, 18 predicted target genes were selected for validation. For *miR-20b*, 10 genes were selected for validation, and for *miR-183*, 11 genes were chosen for validation. Due to space constraints only a select group of the experimentally validated targets are shown here (Fig. 4). Results not presented in Fig. 4 are provided in Additional file 5. Significant reduction in normalized Renilla activity in RCAS-miRNA infected cells relative to RCAS-SC infected cells, confirms miRNA target site recognition. The 3′-UTR of chicken *ADIPOR2* contains binding sites for both *let-7c* and *miR-20b*, both of which were validated in the luciferase reporter assay (Fig. 4a and b). Among its functions, ADIPOR2 controls, in part, the expression of *ACOX1*, which encodes for the first enzyme in fatty acid β-oxidation. Of note, chicken *ACOX1* can also be regulated by *miR-20b* (Fig. 4b). *ELOVL6*, a validated *miR-183* target (Fig. 4c), encodes for a lipogenic enzyme involved in the de novo synthesis of long-chain fatty acids, along with *FASN*, a validated *let-7c* (Fig. 4a) and *miR-183* target (Fig. 4c), and *SCD*, a validated *let-7c* target (Fig. 4a). Among the regulators of *ELOVL6* are *INSIG1*, also a validated *miR-183* target (Fig. 4c) and *SCAP*, a validated *let-7c* target (Fig. 4a). MSMO1 is a sterol-C4-methyl oxidase involved in cholesterol biosynthesis, and we validated a *miR-20b* recognition site within the 3′-UTR of chicken *MSMO1* (Fig. 4b). In silico target prediction indicates that *miR-20b* may regulate multiple genes associated with cholesterol metabolism (Table 2). Other validated miRNA target genes include *FADS1* (*let-7c*, *miR-20b*, and *miR-183*), *FADS2* (*let-7c*), and *SQLE* (*let-7c* and *miR-183*) (Fig. 4). FADS1 and FADS2 are involved in the biosynthesis of unsaturated fatty acids. *SQLE* encodes for the enzyme that catalyzes the first oxygenation step in sterol biosynthesis. Several predicted miRNA targets genes were not experimentally validated as bona fide miRNA target genes. These included *ACAT2* and *CYP51A1* for *let-7c* (Fig. 4a), *ABCD3* and *ACSBG2* for *miR-20b* (Fig. 4b), and *HACD2* for *miR-183* (Fig. 4c).

**Table 2:** Number of predicted Lipid and Carbohydrate metabolic genes regulated by *gga-let-7c*, *gga-miR-183* and *gga-miR-20b*

	Function	Number of predicted target genes
gga-miR-let-7c		
Lipid Metabolism	synthesis of lipid	94
	synthesis of fatty acid	43
	synthesis of prostaglandin E2	22
	synthesis of prostaglandin	26
	synthesis of eicosanoid	31
	synthesis of terpenoid	36
	accumulation of lipid	47
	accumulation of ganglioside	6
	accumulation of sphingolipid	10
	accumulation of glycosphingolipid	9
	concentration of lipid	96
	concentration of phospholipid	28
	concentration of acylglycerol	43
	metabolism of prostaglandin	28
	metabolism of membrane lipid derivative	50
	metabolism of eicosanoid	34
	fatty acid metabolism	68
	biosynthesis of polyunsaturated fatty acids	33
	quantity of steroid	58
	degradation of ganglioside GM1	3
Carbohydrate Metabolism	quantity of carbohydrate	75
	concentration of D-glucose	47
	synthesis of carbohydrate	57
	synthesis of amino sugar	5
	synthesis of N-acetylneuraminic acid	3
	metabolism of carbohydrate	77
	metabolism of polysaccharide	32
	uptake of monosaccharide	39
	uptake of D-glucose	33
	uptake of carbohydrate	40
	transport of monosaccharide	24
	transport of carbohydrate	27
gga-miR-183		
Lipid Metabolism	synthesis of lipid	65
	synthesis of fatty acid	32
	synthesis of phospholipid	20
	synthesis of eicosanoid	22
	synthesis of phosphatidic acid	14
	synthesis of prostaglandin	16
	metabolism of phospholipid	23
	metabolism of eicosanoid	23
	metabolism of membrane lipid derivative	32
	fatty acid metabolism	44
	metabolism of prostaglandin	17
	synthesis of lipid	14
Carbohydrate Metabolism	synthesis of phosphatidic acid	31
	synthesis of carbohydrate	65
gga-miR-20b		
Lipid Metabolism	concentration of phospholipid	17
	concentration of lipid	52
	accumulation of ganglioside GM3	3
	accumulation of steroid	9
	accumulation of cholesterol	7
	synthesis of phospholipid	15
	synthesis of phosphatidic acid	12
	internalization of cholesterol	3
	beta-oxidation of lipid	8
Carbohydrate Metabolism	synthesis of phosphatidic acid	12
	quantity of carbohydrate	34

A predicted target gene may be present in more than one category

### Identification of potential regulatory factors of *gga-miR-20b*

We next sought to identify potential regulatory transcription factors for one of the miRNAs shown to regulate metabolic mRNAs. *MiR-20b* was selected, because its expression was downregulated in the liver upon hatching (Fig. 3b) and because it was confirmed to regulate several upregulated metabolic genes, such as *ADIPOR2*, *FADS1*, and *MSMO1* (Fig. 4b). Potential regulatory proteins controlling expression of *miR-20b* were identified using a yeast-one hybrid system. Approximately 6.2 million colonies were screened, and 48 positive clones, i.e. Aureobasidin A toxin resistance, were sequenced. Potential regulatory proteins, with multiple hits in the library screen of *miR-20b* expression, included ZNF143 (8 clones), HIF1A (14 clones), FOXO3 (14 clones), and EGR1 (12 clones).

## Discussion

In the present study, we identified hundreds of genes that were differentially expressed in the liver during the metabolic transition from embryonic to posthatch development in the chicken. Further, we identified dozens of miRNAs that were differentially expressed during this same period of development and that were predicted to target some of the differentially expressed mRNAs involved in metabolic pathways. Three of these miRNAs were confirmed to regulate levels of their predicted mRNA targets. MiRNAs have been shown to regulate metabolic pathways in the liver of humans and rodents [15, 22–26]. For example, overexpression of *miR-155* in mice reduces liver and serum lipid, triglycerides (**TG**), high density lipoproteins and free fatty acids [27]. *MiR-128-1* and *miR-148a* reduce hepatic levels of proteins involved in lipid trafficking and metabolism and levels of circulating cholesterol and TG [28]. Cholesterol and fatty acid metabolism are regulated by *miR-122* [29]. *MiR-1*, *miR-206*, *miR-613*, and *miR-34a* repress expression of lipogenic genes in the human hepatocyte cell line HepG2 by suppression of nuclear transcription factors such as *LXRα* (*miR-1*, *miR-206*, *miR-613*) [30, 31] and *RXRα* (*miR-34a*) [32]. SREBP-2 regulates cholesterol synthesis and metabolism, while SREBP-1c controls fatty acid synthesis in the liver [33]. *MiR-33*, which is expressed from an intron of the *SREBP*-2 gene, represses expression of *SREBP-1c* in mice [34], and it reduces fatty acid oxidation in human hepatic cells [35]. *MiR-291b-3p* regulates hepatic levels of *SREBP1* and *FASN* mRNA in mice fed a high-fat diet [36]. Overexpression of *miR-185* in HepG2 cells represses *SREBP-2* mRNA [37]. Interestingly, SREBP-1c regulates expression of *miR-185* [38]. Taken together, these findings indicate coordinated regulation of SREBP-2 and SREBP-1c through expression of miRNAs in mammals. *MiR-24* stimulates hepatic lipid accumulation by repressing expression of insulin-induced gene 1 (***INSIG1***) in mice [39]. *MiR-223* inhibits cholesterol biosynthesis by decreasing 3-hydroxy-3-methylglutaryl-CoA synthase 1 (***HMGCS1***) and methylsterol monooxygenase (***MSMO1***) expression [40]. *MiR-34a* regulates hepatic levels of *PPARA* expression, and silencing *miR-34a* increases expression of PPARα-regulated genes [41]. Liver *PPARA* expression is also regulated by *miR-21* and *miR-27b* in humans [42]. These examples show that miRNAs play a critical role in regulating hepatic metabolic pathways in mammals. Our current findings indicate that miRNAs also play critical roles in regulating the hepatic metabolic switch that occurs after hatching in chickens. We found that *miR-20b* was downregulated in the liver upon hatching and that it was predicted to regulate several upregulated metabolic genes, like *ADIPOR2* and *MSMO1* (Figs. 1, 2 and 3). *ADIPOR2* mRNA levels were significantly upregulated (2.7-fold) in the posthatch liver (Fig. 3a). Two miRNAs which were downregulated in the posthatch liver, *let-7c* and *miR-20b*, were predicted to regulate *ADIPOR2* expression (Figs. 1 and 3a). ADIPOR2 controls expression of acyl-Coenzyme A oxidase 1 (***ACOX1***), the first enzyme in fatty acid β-oxidation. ADIPOR2 also mediates several other important metabolic processes, including PPARA activity, and glucose uptake [37]. SREBF1 is a transcription factor that recognizes sterol regulatory element-1 sites and regulates fatty acid and cholesterol synthesis. Hepatic *SREBF1* mRNA levels increased over 18-fold after hatching. SREBF1 is regulated in part by insulin through INSIG1. Levels of *INSIG1* mRNA increased 8-fold after hatching (Fig. 3b). *INSIG1* mRNA is a predicted target of three miRNAs (*miR-182*, *miR-183*, and *miR-454*), all of which decreased 2-fold between D1 and D3 (Figs. 1 and 3b). Levels of *MAP4K4* mRNA, a known inhibitor of lipid metabolism [43], decreased after hatching (Fig. 3c). *MAP4K4* is a predicted target of an upregulated miRNA, *miR-29a* (Fig. 3c). This down-regulation of *MAP4K4* partially accounts for the upregulation of several genes involved in lipid metabolism, including *FASN* and *ACACA*, which are also miRNA-regulated genes (Fig. 3). FASN is responsible for the conversion of acetyl-coA into long-chain fatty acids. *FASN* mRNA levels were 182-fold higher in the posthatch D3 liver compared the E18 liver. *FASN* mRNA is a *miR-107* target (Figs. 2 and 3d). *MiR-107* is also known to function in insulin sensitivity in obese mice [44]. We found *miR-107* to be downregulated in the posthatch liver. Both *ACACA* and *SCD* mRNAs were upregulated in the posthatch liver (42- and 665-fold, respectively). Interestingly, both *ACACA* and *SCD* mRNAs are predicted to be regulated by *miR-200b*, a downregulated miRNA in the posthatch liver (Fig. 2). Another upregulated *miR-200b* target in the posthatch liver is *LPIN1* (Fig. 2). LPIN1 is a phosphohydrolase involved in triglyceride synthesis and is also a transcriptional coactivator of PPARs to modulate lipid metabolic genes [45]. The miRNA *let-7c*, a member of the *let-7* miRNA family, was downregulated in the liver upon hatching (Fig. 3e). *Let-7c* is known to regulate cell proliferation and is often dysregulated in hepatocarcinomas [46]. A major regulator of *let-7c* expression is the transcriptional regulator PPARA [47]. PPARA is a key regulator of lipid metabolism. Interestingly, *let-7c* is a *FADS2* regulator. *FADS2* mRNA levels increased more than 700-fold posthatch (Fig. 3e). FADS2 regulates fatty acid unsaturation [48]. ELOVL2 is involved in fatty acid elongation, and levels of *ELOVL2* mRNA increased 250-fold posthatch (Fig. 3f). *ELOVL2* mRNA is a predicted target of *miR-10b*, which decreased posthatch (Fig. 3f). HMGCS1 is involved in cholesterol synthesis, and levels of *HMGCS1* mRNA increased 30-fold between E18 and D0. *HMGCS1* mRNA is a predicted target of *miR-18a* (Fig. 2), which decreased between E18 and D0 (Fig. 3g). *MSMO1* mRNA was increased 15-fold in the posthatch liver and is targeted by two downregulated miRNAs, *miR-20b* and *miR-454* (Fig. 3h). MSMO1 is a sterol-C4-methyl oxidase involved in cholesterol biosynthesis [49]. The predicted regulation of *ADIPOR2* mRNA by both *let-7c* and *miR-20b* and the regulation of *MSMO1* by *miR-20b* and *FADS2* by *let-7c*, strongly suggests that *let-7c* and *miR-20b* are critical regulators of hepatic metabolic processes during the switch from embryonic to posthatch development.

Further integration of small RNA and mRNA deep sequencing data with IPA pathway analysis and miRNA target prediction, revealed a complex regulatory network consisting of a specific group of miRNAs with overlapping metabolic targets and known metabolic regulatory factors which likely coordinate to facilitate the hepatic metabolic transition in peri-hatch chicks (Additional file 4). Among these are PPARA regulatory pathways (Additional file 4). In the present study, PPARA and many of its target genes exhibited dynamic hepatic expression patterns in late-embryonic/early-posthatch chickens. PPARA is a master regulator of fatty acid oxidation in both mitochondria and peroxisomes (Reviewed by [50]). As discussed above, many of these genes associated with PPARA regulatory pathways are also likely regulated by many of the hepatically-expressed miRNAs identified in the present study (Additional file 4). These miRNAs and their target genes display reciprocal expression during chicken hepatic development (Additional file 4) suggesting that in addition to master transcriptional regulators, such as PPARA, miRNAs are also a major regulatory factor in the metabolic transition in peri-hatching chickens.

Another major transcription regulatory family of lipid metabolic processes is the sterol regulatory element binding proteins (SREBPs) (Reviewed by [51]). SREBP1 (encoded by *SREBF1*) is more involved in the regulation of fatty acid synthesis pathways, while SREBP2 (encoded by *SREBF2*) is more involved in the regulation of cholesterol synthesis (Reviewed by [52]). As discussed above, *SREBF* expression (both *SREBF1* and *SREBF2*) was higher in the livers of post-hatch chickens, than in embryos. Among SREBP1-regulated genes are *MSMO1*, *IDH1*, and *INSIG1*. As mentioned above, MSMO1 is a sterol-C4-methyl oxidase involved in cholesterol biosynthesis. *MSMO1* hepatic expression was barely detectable in the embryonic chick liver with RPKMs of only 11 (E18)-16(D0) then significantly increased by D3 (173 RPKM). Among its functions, INSIG1 regulates SREBP activity by forming a complex with SCAP, which cleaves precursor SREBP proteins into their active forms (Reviewed by [52]). SREBP2-regulated genes include *HMGCR*, *HMGCS1*, and *HMGCS2*. HMGCR activity is the rate limiting step in cholesterol synthesis. HMGCS1 is involved in the production of HMG-CoA, the substrate of HMGCR. *HMGCS1* expression was only slightly expressed in the embryonic chick liver (E18-16 RPKM, E20-73 RPKM) and then increased post-hatching (~400 RPKM-D0-D3). HMGCS2 is also regulated by PPARA and considered as the rate limiting step of ketogenesis (Reviewed by [51]). We found *HMCGS2* was expressed at all developmental time points (average of ~1500 RPKM). Many of these gluconeogenic and cholesterol-associated genes differentially expressed here are regulated by miRNAs identified in this study with inverse expression patterns. This would suggest, as seen with PPARA, that miRNAs also assist in regulation of SREBP-controlled pathways.

In the present study, we confirmed that three miRNAs regulate levels of mRNA for some of their predicted targets. Predicted promoter regions for one of these, *miR-20b*, were used in yeast-one hybrid assays to identify proteins that could regulate its expression. Potential regulatory proteins of *miR-20b* expression included ZNF143, HIF1A, FOXO3, and EGR1. ZNF143 has recently been shown to serve as a chromatin-looping factor by binding directly to promoters and connecting them with distal regulatory elements [53]. HIF1A is a subunit of the heterodimeric hypoxia-inducible factor-1 transcription factor [54]. HIF1A regulates the response to hypoxia and thus is known to activate the transcription of many genes, including glucose transporters and glycolytic enzymes [54]. HIF1A expression was recently shown to increase in the livers of rats fed a diet high in fat [55]. FOXO3 is also associated with the response to hypoxia, as its expression increases under hypoxic conditions [56]. *MiR-421* has been shown to act upstream of FOXO3 in the regulation of lipid metabolism in a nonalcoholic fatty liver disease mouse model [57]. This study further demonstrated that overexpression of *miR-421* reduced FOXO3 protein levels. In our study of the developing chick liver, we found that *miR-421* expression increases upon hatching (2.9-fold), while *FOXO3* expression decreases (3-fold). EGR1 is a regulator of multiple cellular differentiation and proliferation processes [58]. It also regulates hepatic expression of cholesterol biosynthesis genes [59]. In summary, our yeast one-hybrid screening of *miR-20b*, which is associated with the hepatic metabolic switch, revealed that transcription regulators of important metabolic genes also potentially regulate metabolism-associated miRNAs. This suggests that a highly complex and tightly regulated system of molecular mechanisms is governing the metabolic switch that occurs in poultry. For example, increased expression of *miR-421* after hatching might decrease levels of FOXO3, which would lead to decreased expression of *miR-20b*. Decreased levels of *miR-20b* would, in turn, lead to increased expression of FADS1 and MSMO1. Increased levels of FADS1 and MSMO1 would increase fatty acid and cholesterol synthesis, as part of the metabolic switch that occurs after hatching. Similar mechanisms might involve other miRNAs such as *let-7c* and *miR-183*, which we have confirmed can regulate ADIPOR2, ELOVL6, FADS1, FADS2, FASN, HMGCR, LSS, SCD, and SQLE, which also control fatty acid and cholesterol synthesis. Delayed feeding is known to delay the metabolic switch. Expression profiles of miRNAs that regulate the metabolic switch should also shift later in response to delayed feeding. However, effects of delayed feeding on miRNA expression and involvement of miRNA in the delayed feeding response have not been determined. Thus, our novel and extensive data set of the molecular regulatory changes which occur during the metabolic switch, will provide a solid foundation for future studies.

## Conclusion

In summary, we have shown that expression of over 800 mRNAs and 30 miRNAs is altered in the embryonic liver between E18 and D3, and many of these differentially expressed mRNAs and miRNAs are associated with metabolic processes, in particular pathways involved in lipid and cholesterol synthesis. We have confirmed the regulation of some of these mRNAs in metabolic pathways by miRNAs expressed in a reciprocal pattern. Finally, we have identified three proteins that regulate expression of one of these important miRNAs. Integration of the upstream regulatory mechanisms governing miRNA expression along with monitoring the downstream effects of this expression will ultimately allow for the construction of complete miRNA regulatory networks associated with the hepatic metabolic switch in chickens. Understanding which proteins regulate miRNA expression is vital to discovering the underlying molecular regulatory mechanisms of any physiological process. This is particularly true in the case of the metabolic switch in the liver that occurs at hatching of chickens. As miRNAs can quickly and subtly alter multiple pathways at once and this regulation can be fine-tuned by both number of miRNA binding sites as well as the targeting of a single mRNA by multiple miRNA, they represent an ideal regulatory mechanism to govern this process.

## Additional files

Additional file 1: All identified miRNAs expressed in the liver of E18, E20, D0, D1, and D3 chicks. Values are given as CPM. (XLSX 27 kb)

Additional file 2: All identified mRNA transcripts expressed in the liver of E18, E20, D0, D1, and D3 chicks. Values are given as RPKM. (XLSX 4417 kb)

Additional file 3: RT-qPCR confirmation of the hepatic expression patterns of select mRNAs and miRNAs identified by RNA-seq deep sequencing. (PDF 1190 kb)

Additional file 4: Expressional changes in metabolic pathways and their miRNA regulators over the course of late-embryonic/early-posthatch hepatic development in chickens. Green = decreased expression; Red = increased expression; White = steady expression (no change, RPKM > 30); Gray = lowly expressed (RPKM < 30) or not detected. Expression gradient is E18 → D3. (PPTX 4325 kb)

Additional file 5: All chicken target genes for *let-7c*, *miR-20b*, and *miR-183* selected for validation in the RCAS experiment. Relative luciferase activities (relative to the scrambled control) and *p*-values are provided. (XLSX 13 kb)

## Abbreviations

ABCD3: ATP binding cassette subfamily D member 3
ACACA: Acetyl-CoA carboxylase alpha
ACAT2: Acetyl-CoA acetyltransferase two
ACOX1: Acyl-CoA oxidase one
ACSBG2: Acyl-CoA synthase bubblegum family member two
ACSL: Acyl-CoA synthase long-chain
ADIPOR2: Adiponectin receptor two
ALDH3A2: Aldehyde dehydrogenase 3 family member A2
BCAT1: Branched chain amino acid transaminase one
CPM: Counts per million
CYP51A1: Cytochrome P450 family 51 subfamily A member one
D: Posthatch day
E: Embryonic day
EGR1: Early growth response one
EIF: Eukaryotic translation initiation factor
ELOVL: Elongation of very long chain fatty acids
FADS: Fatty acid desaturase
FASN: Fatty acid synthase
FBP: Fructose-biphosphatase
FOXO3: Forkhead box O3
FXR: Farnesoid X receptor
G6PC3: Glucose-6-phosphatase catalytic subunit 3
GH: Growth hormone
GLS2: Glutainase two
GLUD1: Glutamate dehyrogenase one
GPT: Glutamic-pyruvic transaminase
HACD2: 3-hydroxyacyl-CoA dehydratase two
HADHA: Hydroxyaryl-CoA dehydrogenase/3-ketoacyl-CoA thiolase/enoyl-CoA hydratase alpha subunit
HADHB: Hydroxyaryl-CoA dehydrogenase/3-ketoacyl-CoA thiolase/enoyl-CoA hydratase beta subunit
HIF1A: Hypoxia inducible factor one alpha subunit
HMGCR: 3-hydroxy-3-methyl glutaryl-CoA reductase
HMGCS: HMG CoA synthase
HNF4A: Hepatocyte nuclear factor four alpha
HPD: 4-hydroxyphenylpyruvate dioxygenease
IDH1: Isocitrate dehydrogenase (NADP(+)) 1, cytosolic
IL: Interleukin
INSIG1: Insulin-induced gene one
IPA: Ingenuity Pathway Analysis
LPIN1: Lipin one
LPS: Lipopolysaccharide
LXR: Liver X receptor
MAP4K4: Mitogen-activated protein kinase kinase kinase kinase four
ME: Malic enzyme
miRNA: microRNA
mRNA: messenger RNA
MSMO1: Methyl sterol monooxygenase
MYC: V-myc avian myelocytomatosis viral oncogene homolog
PEPCK: Phosphoenolpyruvate carboxkinase
POR: Cytochrome P450 oxidoreductase
PPARA: Peroxisome proliferator-activated receptor alpha
PPARG: Peroxisome proliferator-activated receptor gamma
RCAS: Replication-competent ASLV long terminal repeat with a splice acceptor
RCASBP(A): Replication-competent, ALV LTR promoters, splice acceptor, Bryan-strain pol gene, subgroup A
RCAS-SC: RCAS-scrambled control
RNAi: RNA interference
RPKM: Reads per kilobase per million mapped reads
RXR: Retinoid X receptor
SCAP: SREBF chaperone
SCD: Steraoyl-Coenzyme A desaturase
SPF: Specific pathogen free
SQLE: Squalene epoxidase
SREBF: Sterol regulatory element-binding transcription factor
SREBP: Sterol regulatory element-binding protein
TG: Triglycerides
TP53: Tumor protein P53
XBP1: X-box binding protein one
ZNF143: Zinc finger protein 143
