# Supplementary material for: Identification of microRNAs controlling hepatic mRNA levels for metabolic genes during the metabolic transition from embryonic to posthatch development in the chicken
Source: BMC Genomics. 2017 Sep 5;18:687. doi: 10.1186/s12864-017-4096-5 (PMC5583987; doi:10.1186/s12864-017-4096-5)
Supplement: Supplementary file 3 — RT-qPCR confirmation of the hepatic expression patterns of select mRNAs and miRNAs identified by RNA-seq deep sequencing. (PDF 1190 kb) [file 12864_2017_4096_MOESM3_ESM.pdf]

Additional File 3. RT-qPCR confirmation of the hepatic expression patterns of select mRNAs and miRNAs identified by RNA-seq deep sequencing.

Methods

We utilized RT-qPCR to confirm both the small RNA-seq and RNA-seq deep sequencing results. Total RNA was isolated using Tri-Reagent (Sigma), following the manufacturer's instructions, with the exception that the RNA was precipitated overnight at -20°C. Total RNA was DNase-treated using a TURBO-DNA-free kit (Life Technologies), following the manufacturer's instructions. Total RNA (1µg) from four individual birds at each time point (E18, E20, D0, D1, and D3) was reverse transcribed using a miScript II RT kit (Qiagen) according to the manufacturer's instructions. For mRNAs, forward and reverse primers were designed using primer-BLAST (https://www.ncbi.nlm.nih.gov/tools/primer-blast/) and for miRNAs, the forward primer consists of the mature miRNA sequence and the reverse primer used was the miScript universal primer (Qiagen). Ribosomal protein L4 (*RPL4*) and the small nucleolar RNA, *snoU83B*, were used as housekeeping genes for mRNA and miRNA normalization, respectively. All primer sequences are provided in the table below. For small RNA expression analysis each reaction contained 10 ng of cDNA, 500 nmol of gene-specific forward primer, 1X Universal miScript primer (Qiagen) and 1X iQ SYBR Green Supermix (Bio-Rad). The following PCR conditions were used: 95°C for 5 minutes, followed by 40 cycles of 95°C for 10 seconds, then 58°C for 20 seconds. All reactions were performed in duplicate. Gene specific amplification was confirmed using melting curve analysis. The same conditions were also used for gene expression (mRNA) analysis with the exception that 500 nmol of both a gene-specific forward primer and a gene-specific reverse primer was used. Threshold cycle (Ct) values normalized to the expression levels of *snoU83B* (for miRNAs) or *RPL4* (mRNAs).

Sequences of primers used for qRT-PCR	
Primer	Sequence 5′→3′
ACACA-forward primer	TGGCAGCCATGTTCAGAGAG
ACACA-reverse primer	GGAAATTCCCTCTTCTGTGCCA
ELOVL2-forward primer	TGCTCATAGAGCTCATCCTTGC
ELOVL2-reverse primer	CATAGCACCTTGGCTACCCG
FADS1-forward primer	ACGCCACGGATCCTTTCATAG
FADS1-reverse primer	AAGCTGGGTTGATCCGGTG
FADS2-forward primer	TCTTAATGGGGAGGGAACAGGT
FADS2-reverse primer	ACCAGCTTCTTGCATTTTCACA
FASN-forward primer	GGCTACACACTAGTTGGCACT
FASN-reverse primer	CACTGTGTTCCCATGCCTGA
HADHA-forward primer	GCCGCCGCTAAGTATTGCC
HADHA-reverse primer	CACGTGGGTTCTAGCCTGA
HMGCS1-forward primer	CAGTTCTTGGGATGGACGCT
HMGCS1-reverse primer	GCATTTGACCCAACTAGCATAGC
INSIG1-forward primer	TGGTGTTTATCAGTATACGTCCCC
INSIG1-reverse primer	AGCTGGCGTCCTATGTTTCC
MAP4K4-forward primer	GCAGGCCATGTTACTGGAATAC
MAP4K4-reverse primer	TGACAGGAGGTAGGCTTGCT
MSMO1-forward primer	CATCCTTGGAGCTGGCTTCTT
MSMO1-reverse primer	TCATAGCCACTGTGCACATCAA
PPARA-forward primer	ATAACAGACACCCTTTCACCAGC
PPARA-reverse primer	TGTTTTCTGCTCCACTGGGT
SCD-forward primer	ACGCAAACACCCAGATGTCA
SCD-reverse primer	GAGGGCTTGTAGTATCTCCGC
SREBF1-forward primer	CTCAGCGACATCGACGACAT
SREBF1-reverse primer	GACACGGTGCTGTCAGGG
SREBF2-forward primer	GTGCCGAAGAGAAGATCCCG
SREBF2-reverse primer	AGGAAATAGCTCGCCAGGAAG
RPL4-forward primer	GCACCGCAGAGTGAACGTAA
RPL4-reverse primer	CTCCTCAATGCGGTGACCTTT
miR-20b-forward primer	CAAAGTGCTCATAGTGCAGGTAG
let-7c-forward primer	TGAGGTAGTAGGTTGTATGGTT
miR-183-forward primer	TATGGCACTGGTAGAATTCACTG
miR-15a-forward primer	TAGCAGCACATAATGGTTTGT
miR-454-forward primer	TAGTGCAATATTGCTTATAGGGT
miR-29a-forward primer	TAGCACCATTTGAAATCGGTT
miR-107-forward primer	AGCAGCATTGTACAGGGCTATCA
miR-10b-forward primer	TACCCTGTAGAACCGAATTTGT
miR-18a-forward primer	TAAGGTGCATCTAGTGCAGATA
snoU83B-forward primer	ACCATGGAATAAGCGCTGGGCA

Results

The hepatic expression patterns of 17 mRNAs and nine miRNAs, identified in the RNA-seq experiments were evaluated using RT-qPCR. As shown in the figure below the expression patterns of the analyzed miRNAs and mRNAs were consistent between the two independent quantification methods, deep sequencing and RT-qPCR, demonstrating the validity of the deep sequencing results.

Figure: RT-qPCR validation of select miRNAs and mRNAs identified in RNA-seq deep sequencing of the developing chick liver. RNA-seq data (black lines) are presented as either $\log_2$ CPM (miRNAs) or $\log_2$ RPKM (mRNAs) on the right vertical axes. The RT-qPCR results are presented as the average (4 birds) delta Ct for each developmental time point on the left axes. For miRNAs (blue lines), expression was normalized to the expression levels of *snoU83B* and for mRNAs (green lines) expression was normalized to the expression levels of *RPL4*.

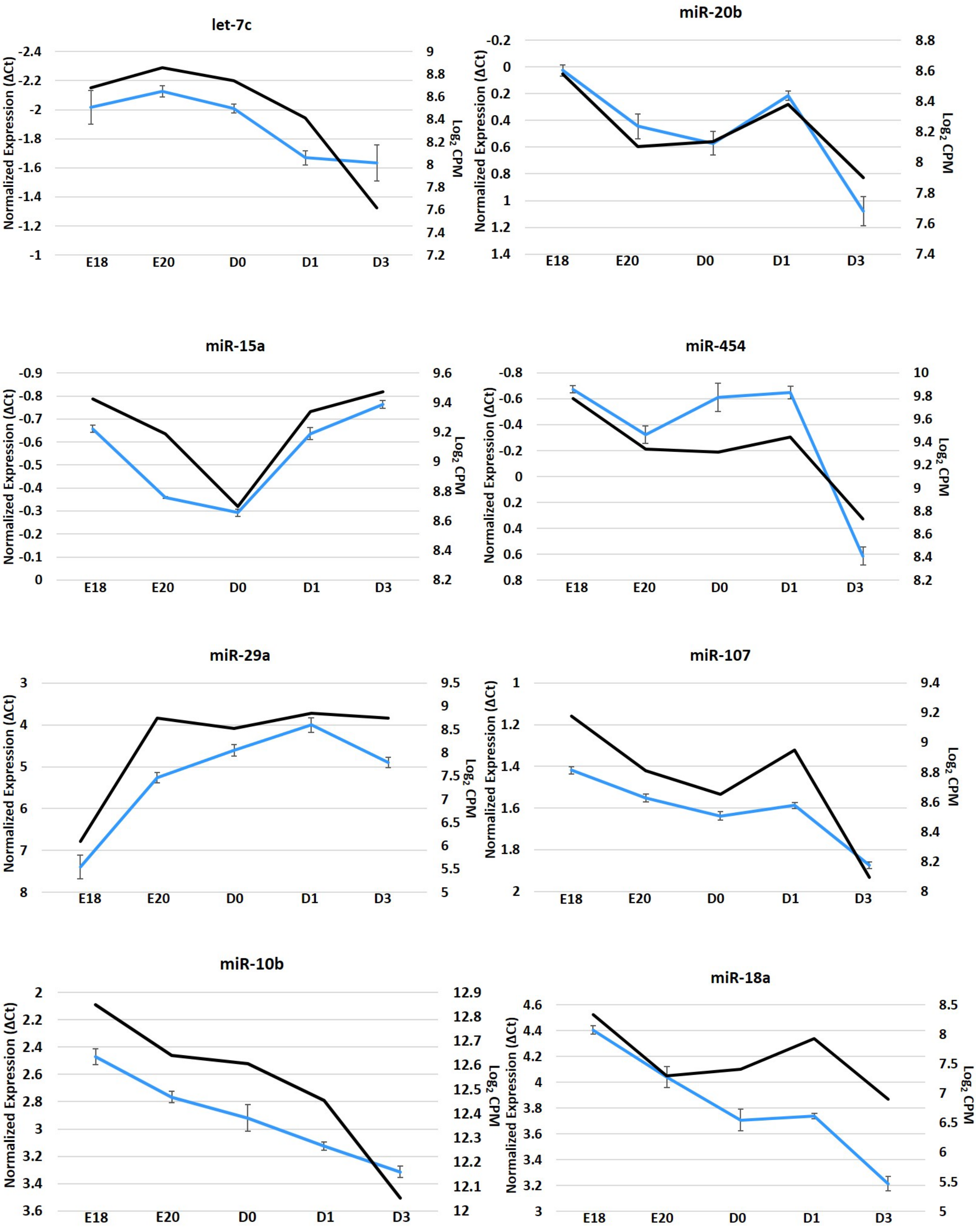

let-7c
Normalized Expression (ΔCt)
-2.4
-2.2
-2
-1.8
-1.6
-1.4
-1.2
-1
Log2 CPM
9
8.8
8.6
8.4
8.2
8
7.8
7.6
7.4
7.2
E18
E20
D0
D1
D3
miR-20b
Normalized Expression (ΔCt)
-0.2
0
0.2
0.4
0.6
0.8
1
1.2
1.4
Log2 CPM
8.8
8.6
8.4
8.2
8
7.8
7.6
7.4
E18
E20
D0
D1
D3
miR-15a
Normalized Expression (ΔCt)
-0.9
-0.8
-0.7
-0.6
-0.5
-0.4
-0.3
-0.2
-0.1
0
Log2 CPM
9.6
9.4
9.2
9
8.8
8.6
8.4
8.2
E18
E20
D0
D1
D3
miR-454
Normalized Expression (ΔCt)
-0.8
-0.6
-0.4
-0.2
0
0.2
0.4
0.6
0.8
Log2 CPM
10
9.8
9.6
9.4
9.2
9
8.8
8.6
8.4
8.2
E18
E20
D0
D1
D3
miR-29a
Normalized Expression (ΔCt)
3
4
5
6
7
8
Log2 CPM
9.5
9
8.5
8
7.5
7
6.5
6
5.5
5
E18
E20
D0
D1
D3
miR-107
Normalized Expression (ΔCt)
1
1.2
1.4
1.6
1.8
2
Log2 CPM
9.4
9.2
9
8.8
8.6
8.4
8.2
8
E18
E20
D0
D1
D3
miR-10b
Normalized Expression (ΔCt)
2
2.2
2.4
2.6
2.8
3
3.2
3.4
3.6
Log2 CPM
12.9
12.8
12.7
12.6
12.5
12.4
12.3
12.2
12.1
12
E18
E20
D0
D1
D3
miR-18a
Normalized Expression (ΔCt)
4.6
4.4
4.2
4
3.8
3.6
3.4
3.2
3
Log2 CPM
8.5
8
7.5
7
6.5
6
5.5
5
E18
E20
D0
D1
D3

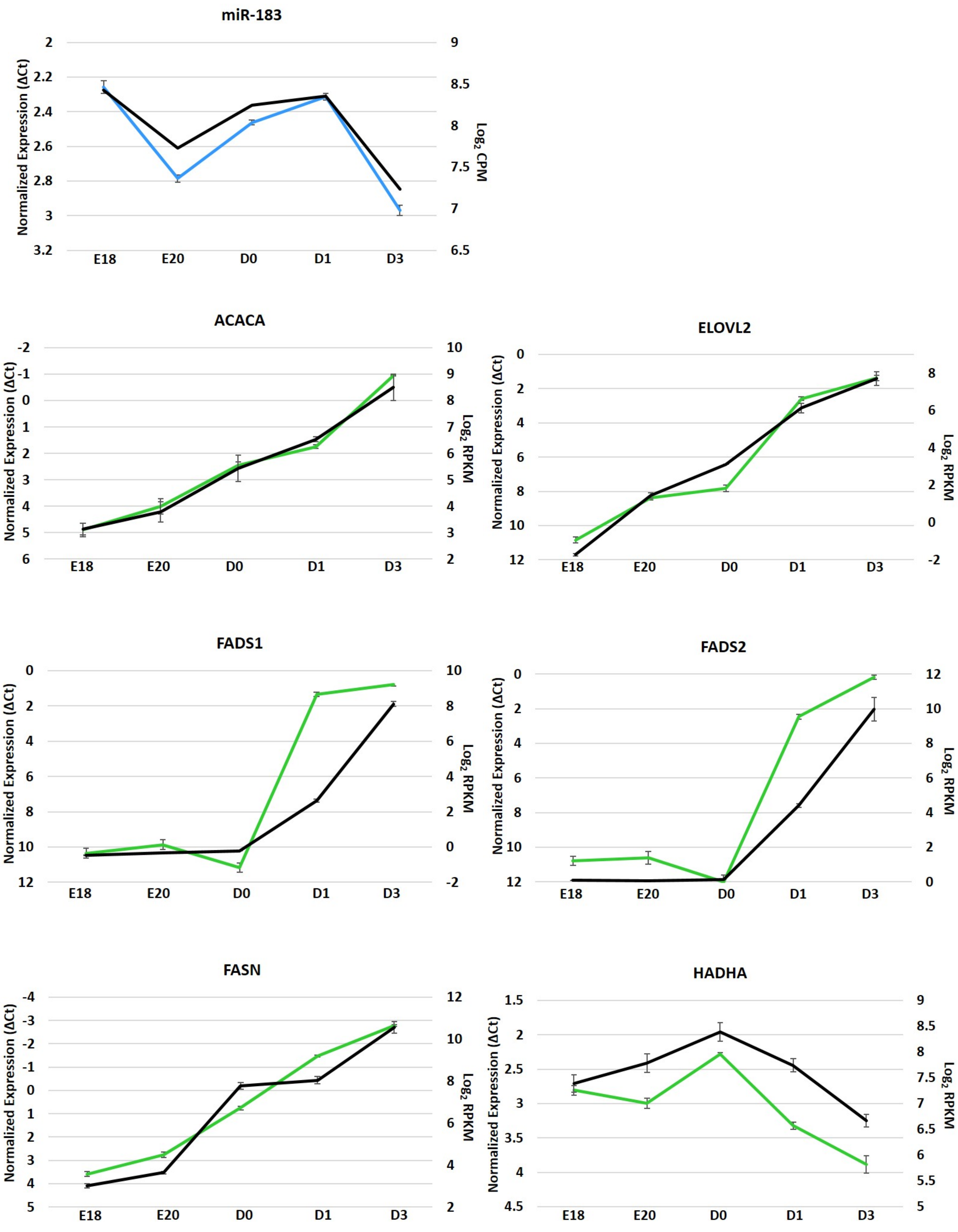
miR-183
Normalized Expression (ΔCt)
Log2 CPM
2
2.2
2.4
2.6
2.8
3
3.2
9
8.5
8
7.5
7
6.5
E18
E20
D0
D1
D3
ACACA
Normalized Expression (ΔCt)
Log2 RPKM
-2
-1
0
1
2
3
4
5
6
10
9
8
7
6
5
4
3
2
E18
E20
D0
D1
D3
ELOVL2
Normalized Expression (ΔCt)
Log2 RPKM
0
2
4
6
8
10
12
8
6
4
2
0
-2
E18
E20
D0
D1
D3
FADS1
Normalized Expression (ΔCt)
Log2 RPKM
0
2
4
6
8
10
12
10
8
6
4
2
0
-2
E18
E20
D0
D1
D3
FADS2
Normalized Expression (ΔCt)
Log2 RPKM
0
2
4
6
8
10
12
12
10
8
6
4
2
0
E18
E20
D0
D1
D3
FASN
Normalized Expression (ΔCt)
Log2 RPKM
-4
-3
-2
-1
0
1
2
3
4
5
12
10
8
6
4
2
E18
E20
D0
D1
D3
HADHA
Normalized Expression (ΔCt)
Log2 RPKM
1.5
2
2.5
3
3.5
4
4.5
9
8.5
8
7.5
7
6.5
6
5.5
5
E18
E20
D0
D1
D3

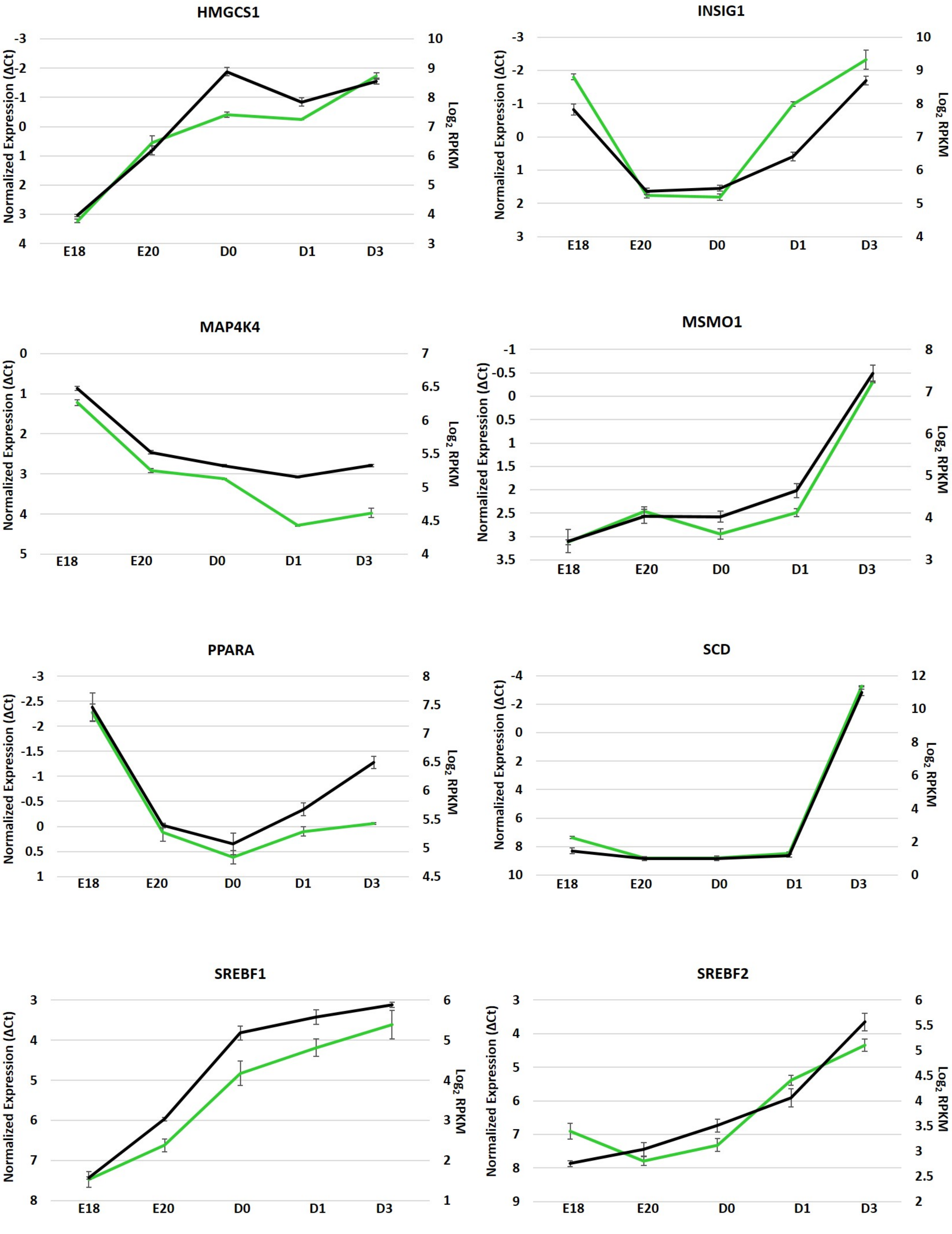
HMGCS1
Normalized Expression (ΔCt)
-3
-2
-1
0
1
2
3
4
Log2 RPKM
10
9
8
7
6
5
4
3
E18
E20
D0
D1
D3
INSIG1
Normalized Expression (ΔCt)
-3
-2
-1
0
1
2
3
Log2 RPKM
10
9
8
7
6
5
4
E18
E20
D0
D1
D3
MAP4K4
Normalized Expression (ΔCt)
0
1
2
3
4
5
Log2 RPKM
7
6.5
6
5.5
5
4.5
4
E18
E20
D0
D1
D3
MSMO1
Normalized Expression (ΔCt)
-1
-0.5
0
0.5
1
1.5
2
2.5
3
3.5
Log2 RPKM
8
7
6
5
4
3
E18
E20
D0
D1
D3
PPARA
Normalized Expression (ΔCt)
-3
-2.5
-2
-1.5
-1
-0.5
0
0.5
1
Log2 RPKM
8
7.5
7
6.5
6
5.5
5
4.5
E18
E20
D0
D1
D3
SCD
Normalized Expression (ΔCt)
-4
-2
0
2
4
6
8
10
Log2 RPKM
12
10
8
6
4
2
0
E18
E20
D0
D1
D3
SREBF1
Normalized Expression (ΔCt)
3
4
5
6
7
8
Log2 RPKM
6
5
4
3
2
1
E18
E20
D0
D1
D3
SREBF2
Normalized Expression (ΔCt)
3
4
5
6
7
8
9
Log2 RPKM
6
5.5
5
4.5
4
3.5
3
2.5
2
E18
E20
D0
D1
D3
