# Supplementary material for: Identification of microRNAs controlling hepatic mRNA levels for metabolic genes during the metabolic transition from embryonic to posthatch development in the chicken
Source: BMC Genomics. 2017 Sep 5;18:687. doi: 10.1186/s12864-017-4096-5 (PMC5583987; doi:10.1186/s12864-017-4096-5)

## Slide 1
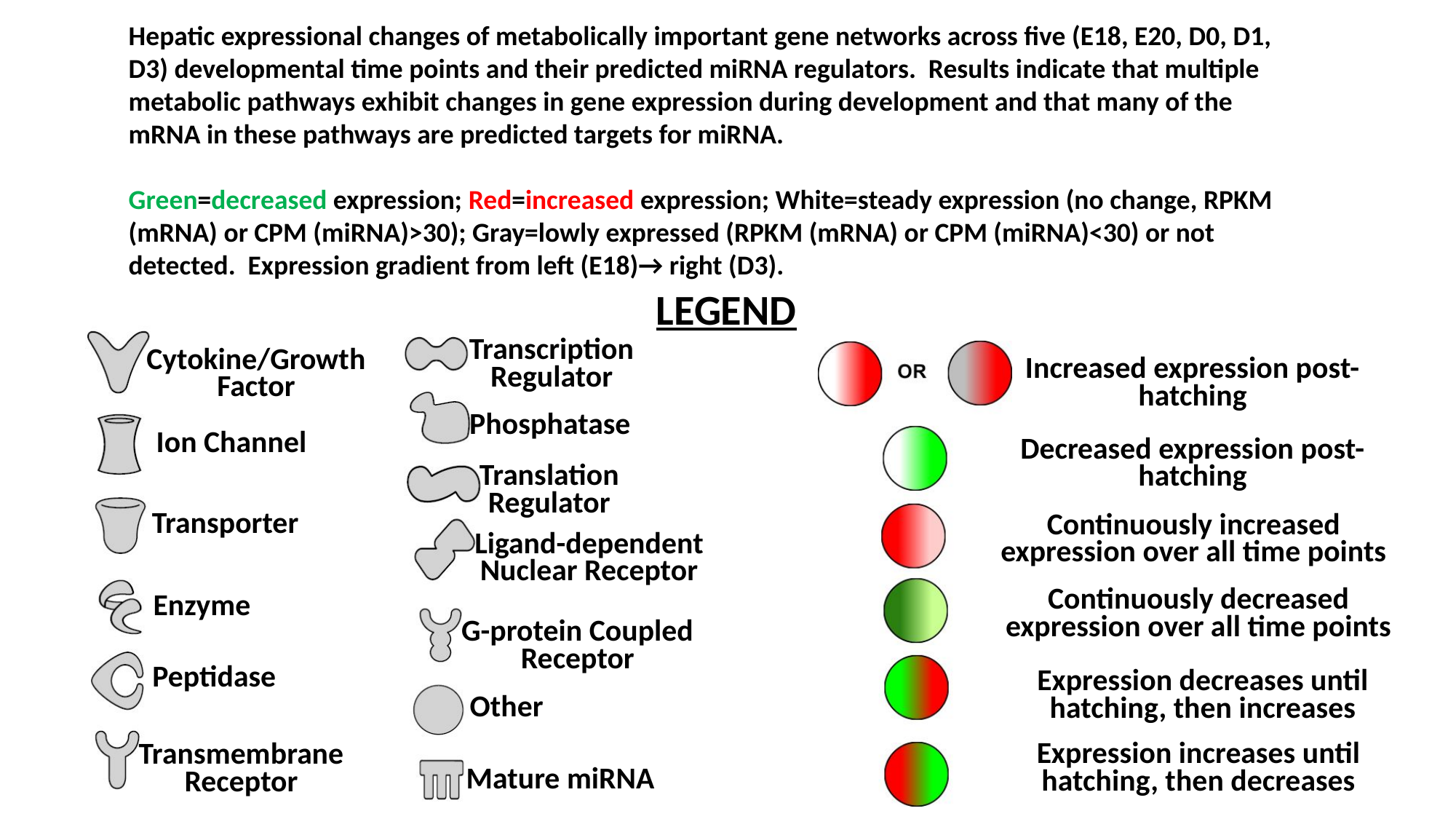

Hepatic expressional changes of metabolically important gene networks across five (E18, E20, D0, D1, D3) developmental time points and their predicted miRNA regulators. Results indicate that multiple metabolic pathways exhibit changes in gene expression during development and that many of the mRNA in these pathways are predicted targets for miRNA.
Green=decreased expression; Red=increased expression; White=steady expression (no change, RPKM (mRNA) or CPM (miRNA)>30); Gray=lowly expressed (RPKM (mRNA) or CPM (miRNA)<30) or not detected. Expression gradient from left (E18)→ right (D3).
LEGEND
Transcription Regulator
Cytokine/Growth Factor
Phosphatase
Ion Channel
Translation Regulator
Transporter
Ligand-dependent Nuclear Receptor
Enzyme
G-protein Coupled Receptor
Peptidase
Other
Transmembrane Receptor
Mature miRNA
Increased expression post-hatching
Decreased expression post-hatching
Continuously increased expression over all time points
Continuously decreased expression over all time points
Expression decreases until hatching, then increases
Expression increases until hatching, then decreases

## Slide 2
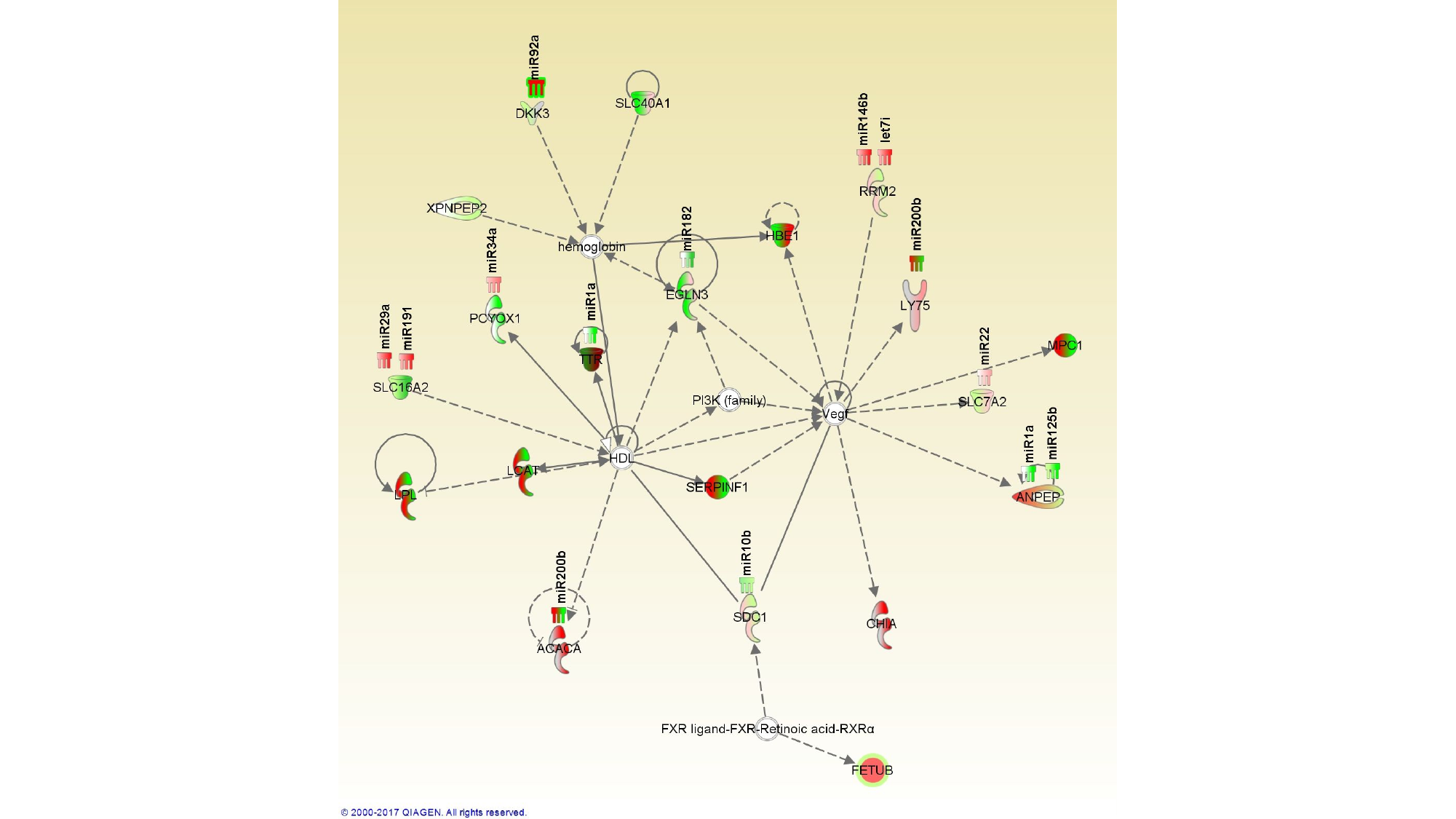

## Slide 3
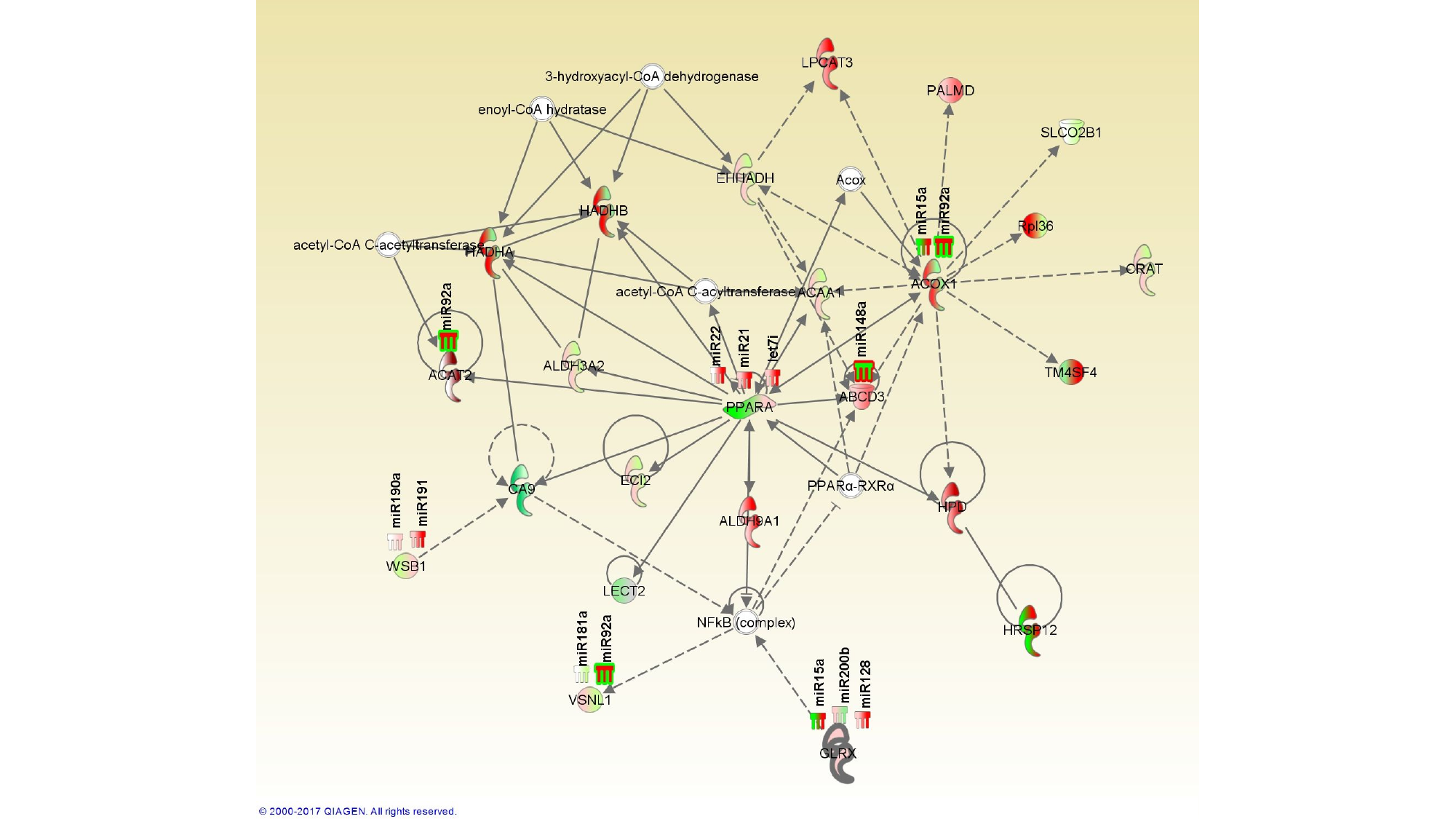

## Slide 4
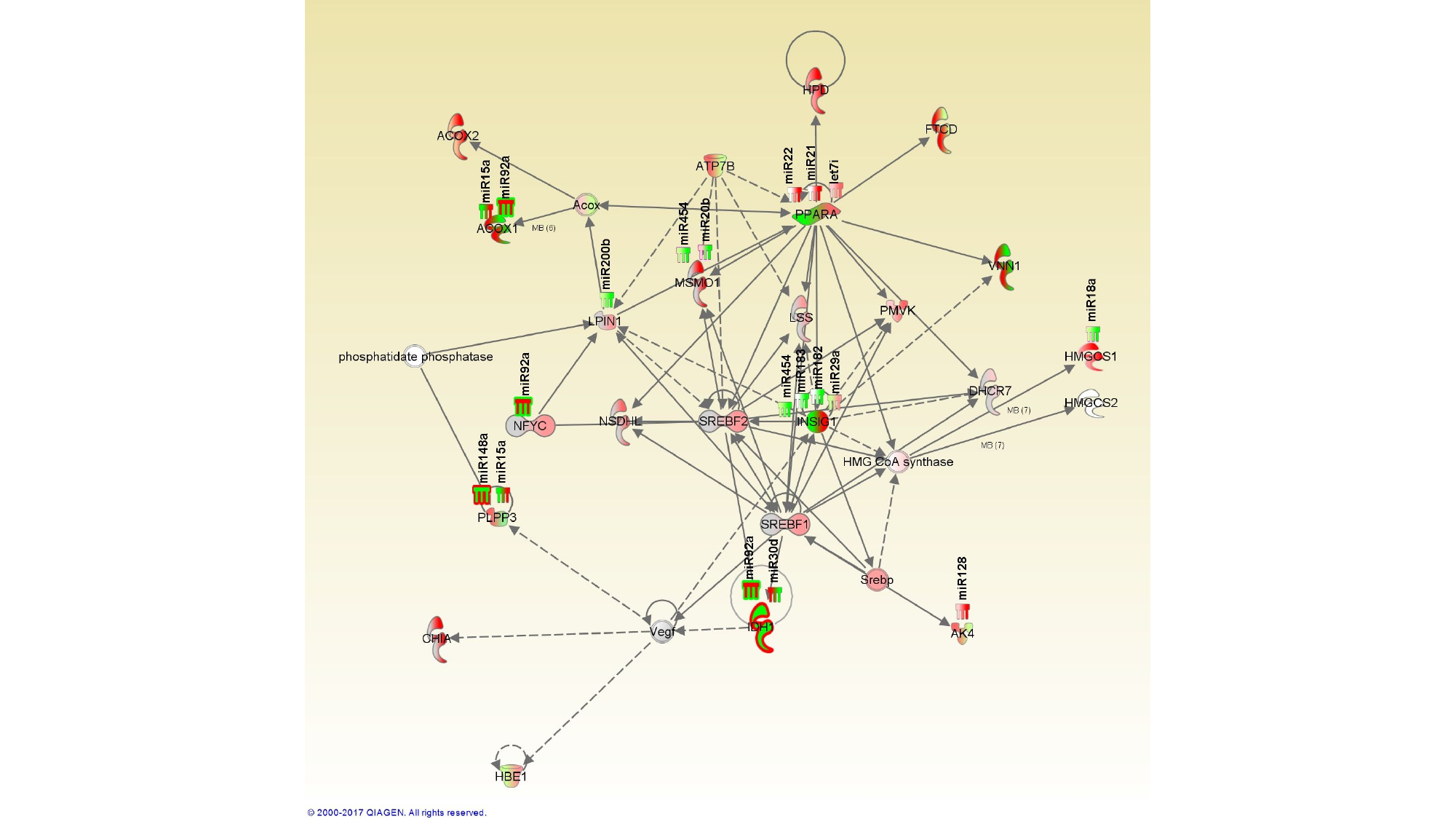

## Slide 5
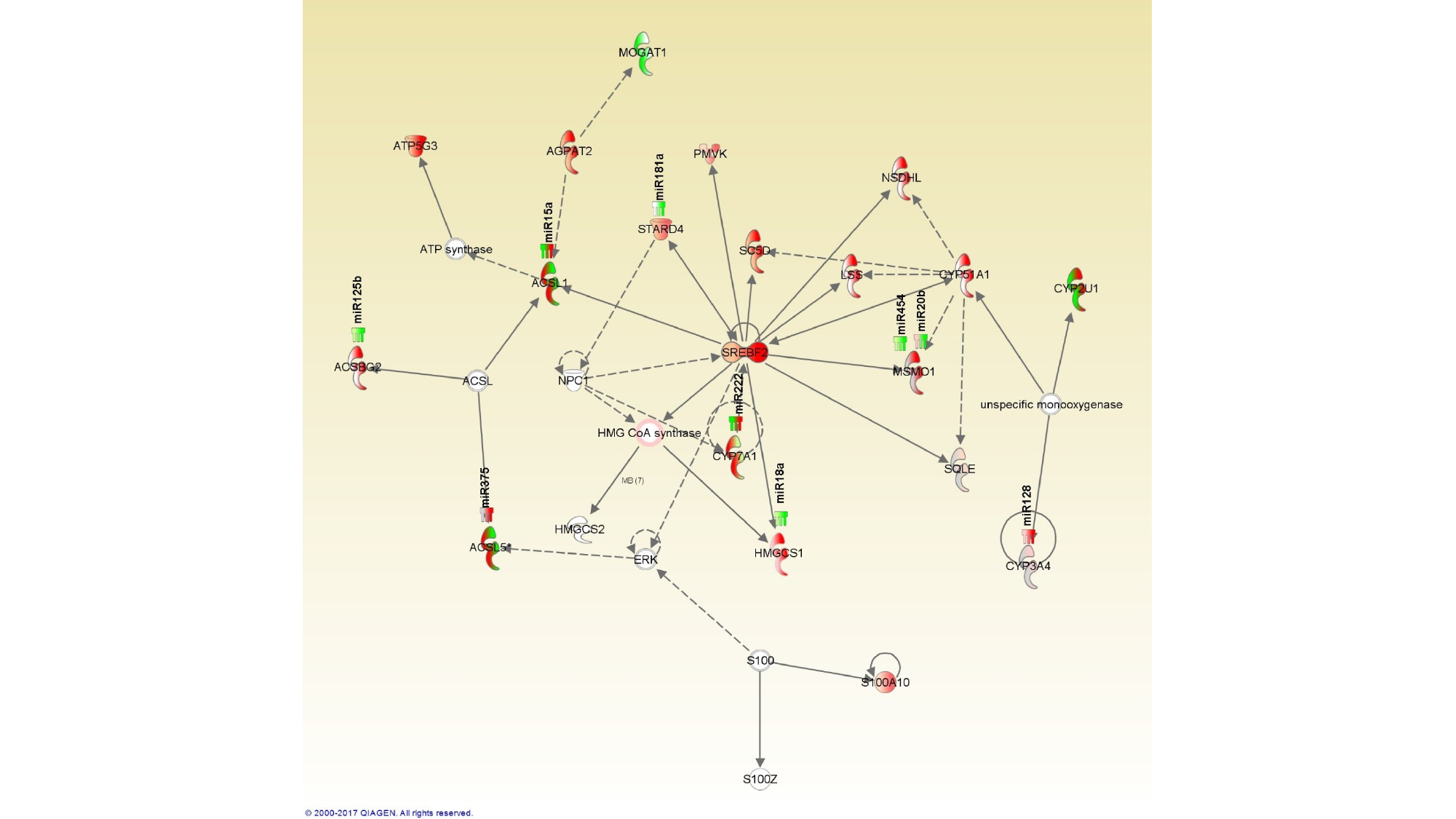

## Slide 6
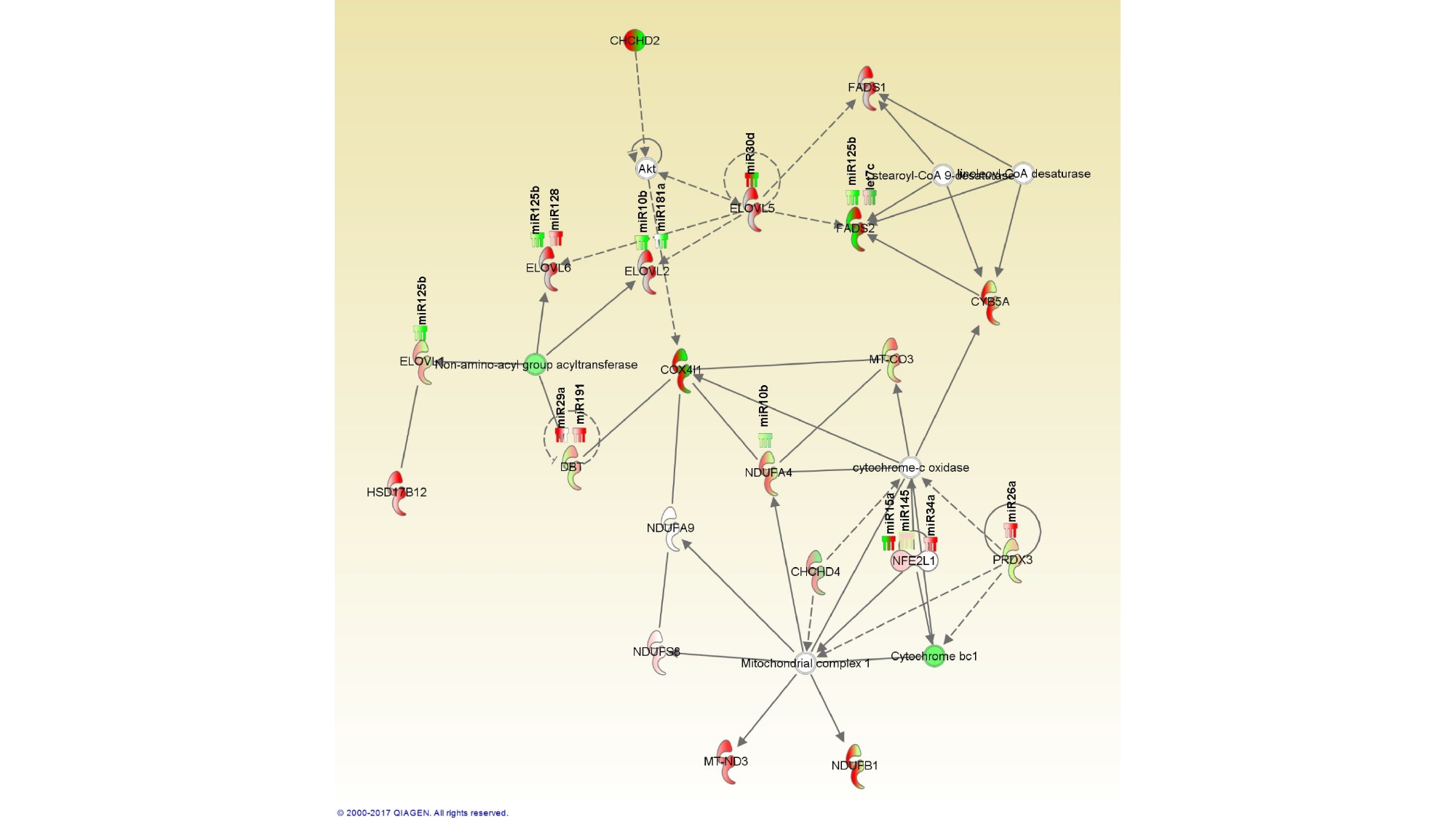

## Slide 7
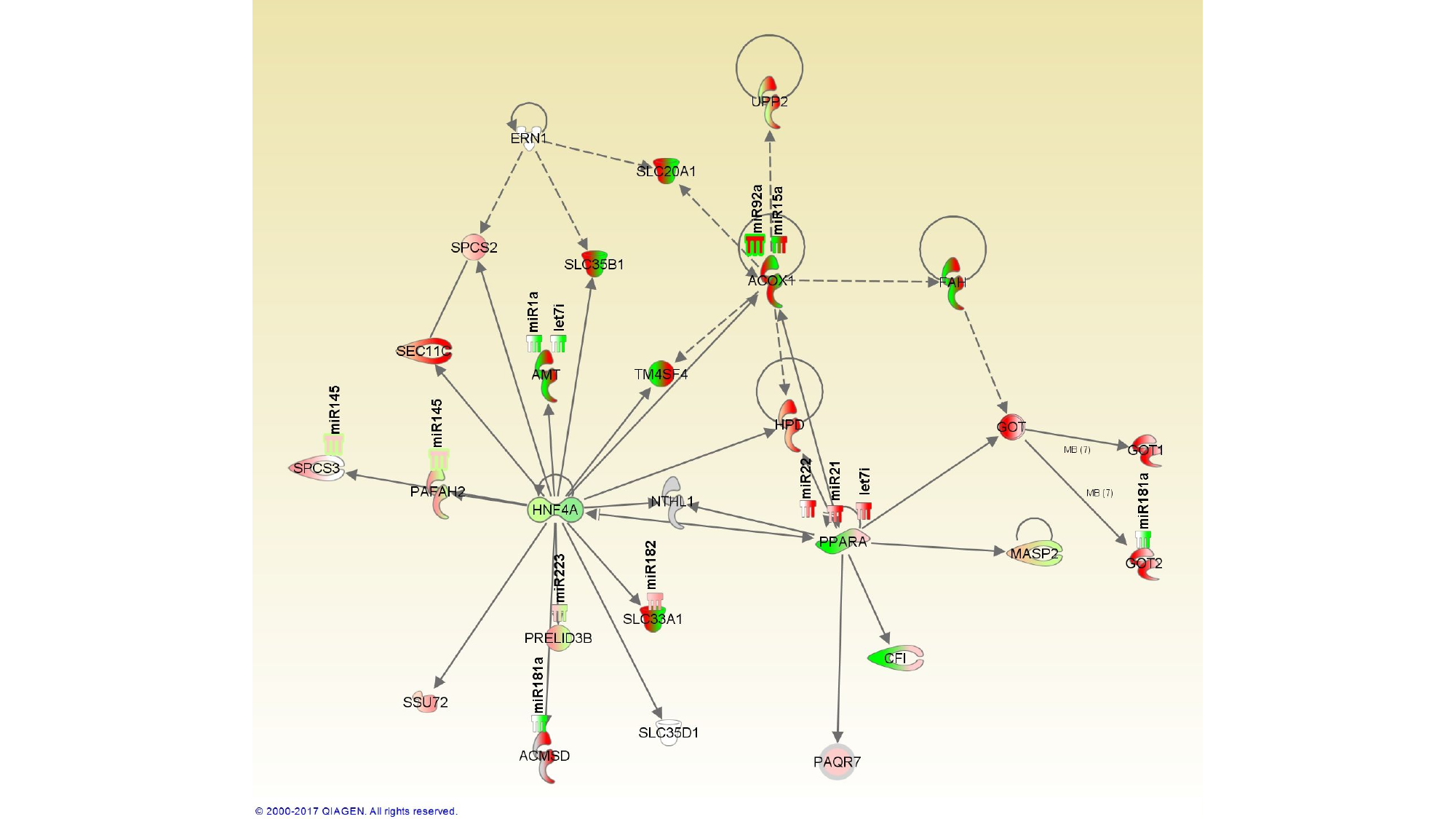

## Slide 8
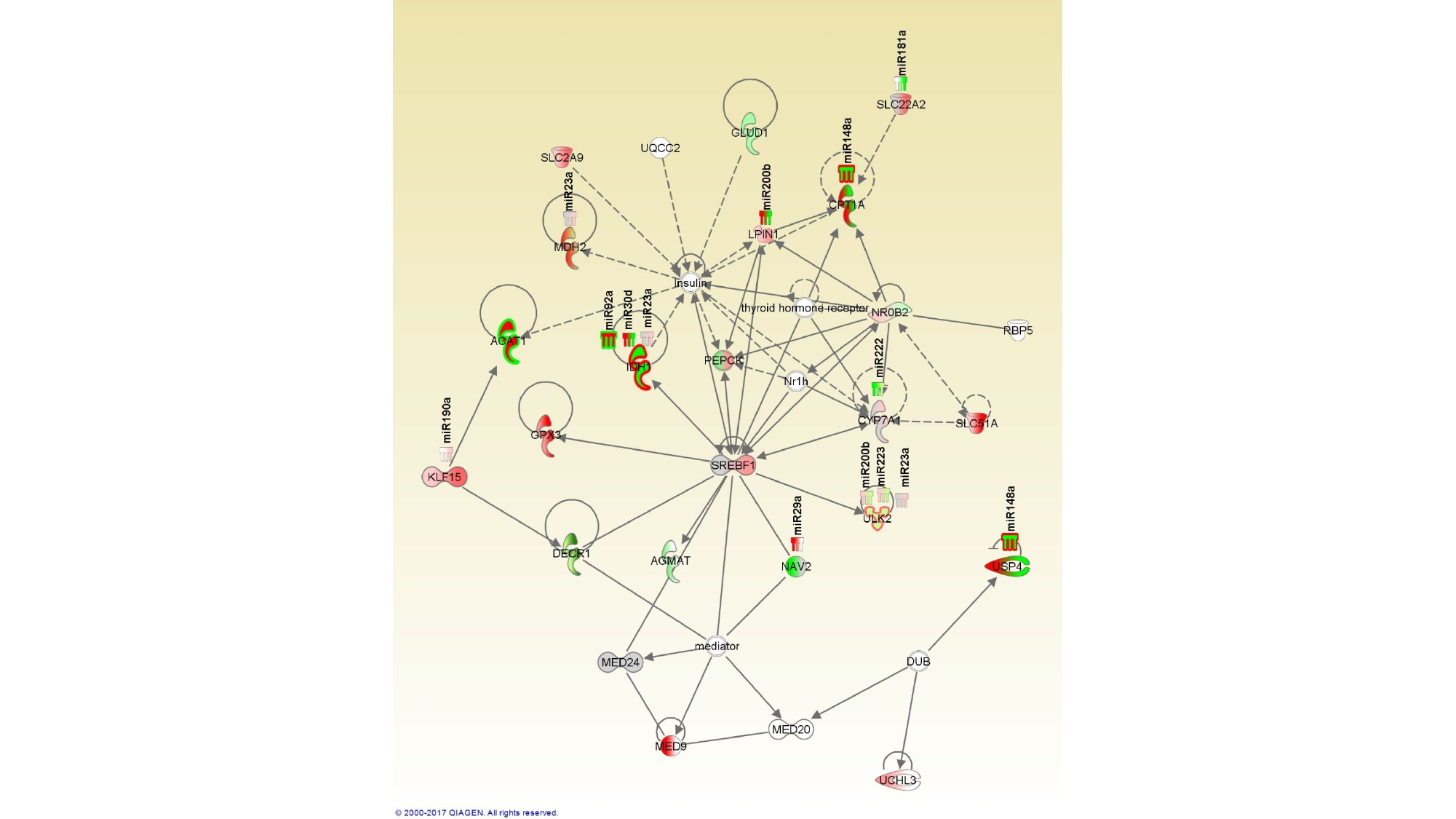

## Slide 9
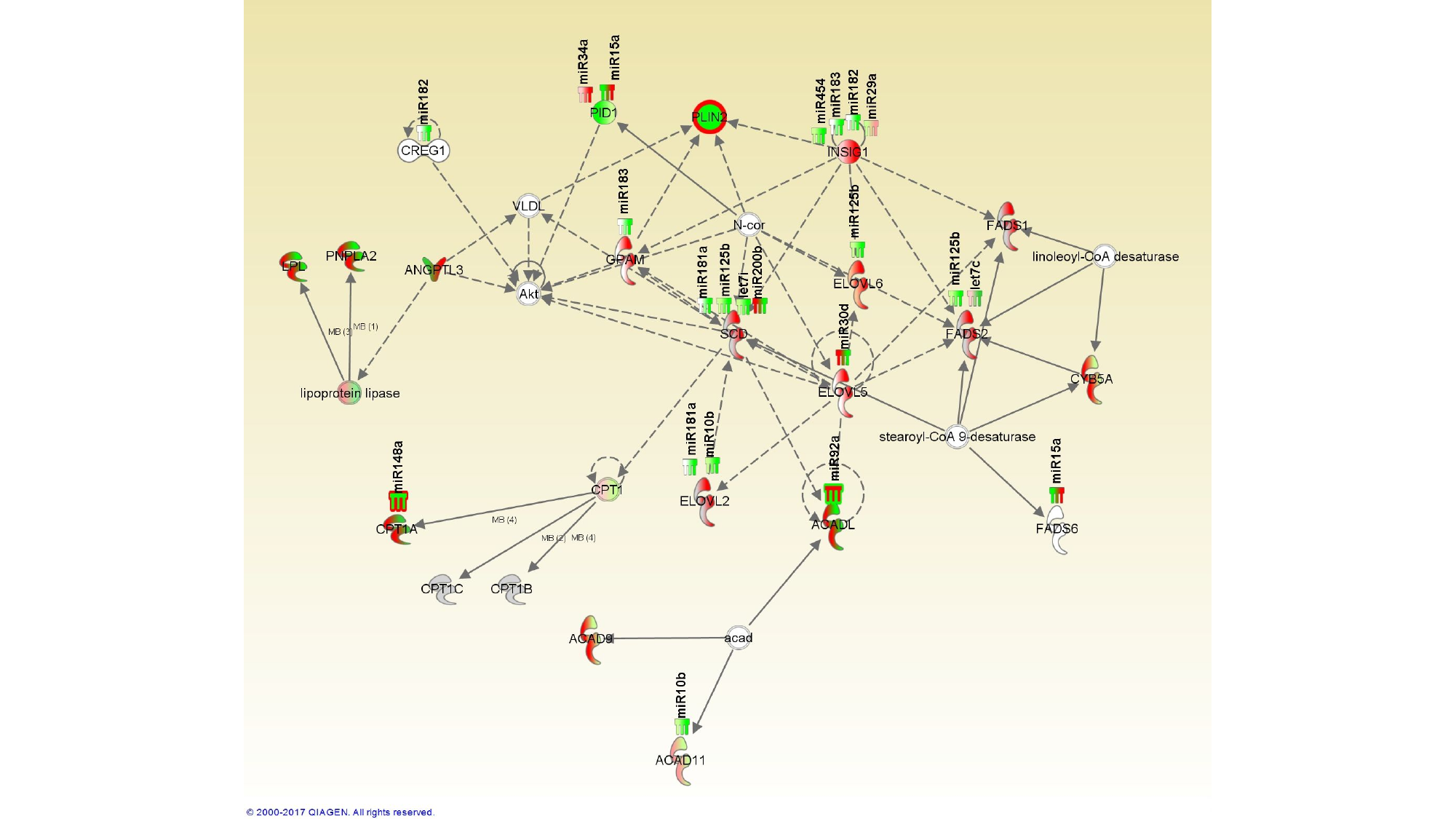

## Slide 10
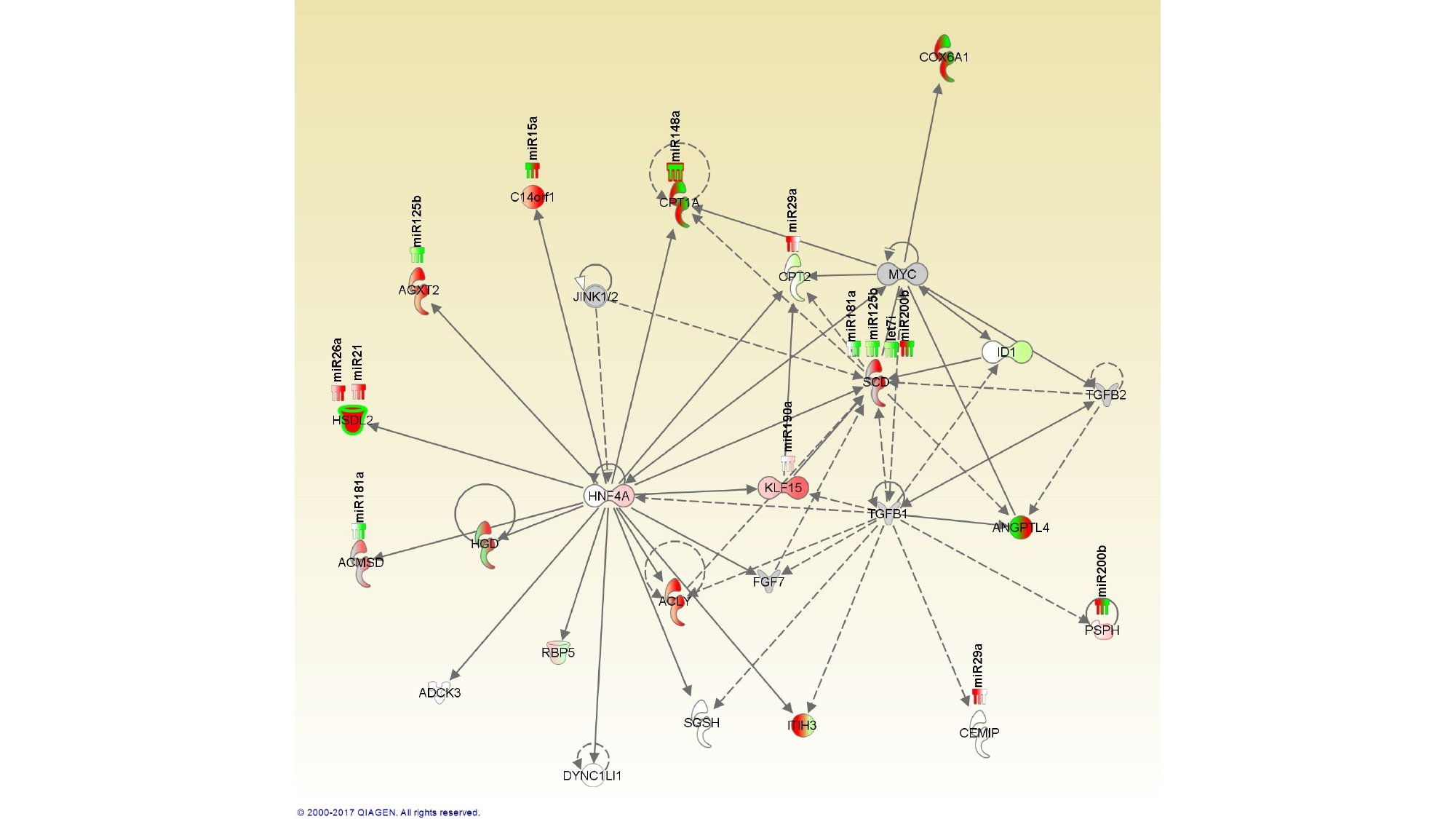

Supplement: Supplementary file 4 — Expressional changes in metabolic pathways and their miRNA regulators over the course of late-embryonic/early-posthatch hepatic development in chickens. Green = decreased expression; Red = increased expression; White = steady expression (no change, RPKM > 30); Gray = lowly expressed (RPKM < 30) or not detected. Expression gradient is E18 → D3. (PPTX 4325 kb) [file 12864_2017_4096_MOESM4_ESM.pptx]
